# Comprehensive review of CRISPR-based gene editing: mechanisms, challenges, and applications in cancer therapy

**DOI:** 10.1186/s12943-023-01925-5

**Published:** 2024-01-09

**Authors:** Mohammad Chehelgerdi, Matin Chehelgerdi, Milad Khorramian-Ghahfarokhi, Marjan Shafieizadeh, Esmaeil Mahmoudi, Fatemeh Eskandari, Mohsen Rashidi, Asghar Arshi, Abbas Mokhtari-Farsani

**Affiliations:** 1 Novin Genome (NG) Lab, Research and Development Center for Biotechnology, Shahrekord, Iran; 2 Young Researchers and Elite Club, Shahrekord Branch, Islamic Azad University, Shahrekord, Iran; 3 Division of Biotechnology, Department of Pathobiology, School of Veterinary Medicine, Shiraz University, Shiraz, Iran; 4 Department of Chemistry, Shahid Bahonar University of Kerman, Kerman, Iran; 5 Faculty of Molecular and Cellular Biology -Genetics, Islamic Azad University of Falavarjan, Isfahan, Iran; 6 Department Pharmacology, Faculty of Medicine, Mazandaran University of Medical Sciences, Sari, Iran; 7 The Health of Plant and Livestock Products Research Center, Mazandaran University of Medical Sciences, Sari, Iran; 8 Young Researchers and Elite Club, Najafabad Branch, Islamic Azad University, Najafabad, Iran; 9 Department of Biology, Nourdanesh Institute of Higher Education, Meymeh, Isfahan Iran

**Keywords:** CRISPR system, Genome editing, Cancer therapy, Genetic mutations, Tumor growth, Immune response, Preclinical studies, Clinical trials, Safety, Delivery, Off-target effects, Cancer-killing molecules

## Abstract

The CRISPR system is a revolutionary genome editing tool that has the potential to revolutionize the field of cancer research and therapy. The ability to precisely target and edit specific genetic mutations that drive the growth and spread of tumors has opened up new possibilities for the development of more effective and personalized cancer treatments. In this review, we will discuss the different CRISPR-based strategies that have been proposed for cancer therapy, including inactivating genes that drive tumor growth, enhancing the immune response to cancer cells, repairing genetic mutations that cause cancer, and delivering cancer-killing molecules directly to tumor cells. We will also summarize the current state of preclinical studies and clinical trials of CRISPR-based cancer therapy, highlighting the most promising results and the challenges that still need to be overcome. Safety and delivery are also important challenges for CRISPR-based cancer therapy to become a viable clinical option. We will discuss the challenges and limitations that need to be overcome, such as off-target effects, safety, and delivery to the tumor site. Finally, we will provide an overview of the current challenges and opportunities in the field of CRISPR-based cancer therapy and discuss future directions for research and development. The CRISPR system has the potential to change the landscape of cancer research, and this review aims to provide an overview of the current state of the field and the challenges that need to be overcome to realize this potential.

## Introduction

**Fig. 1:**
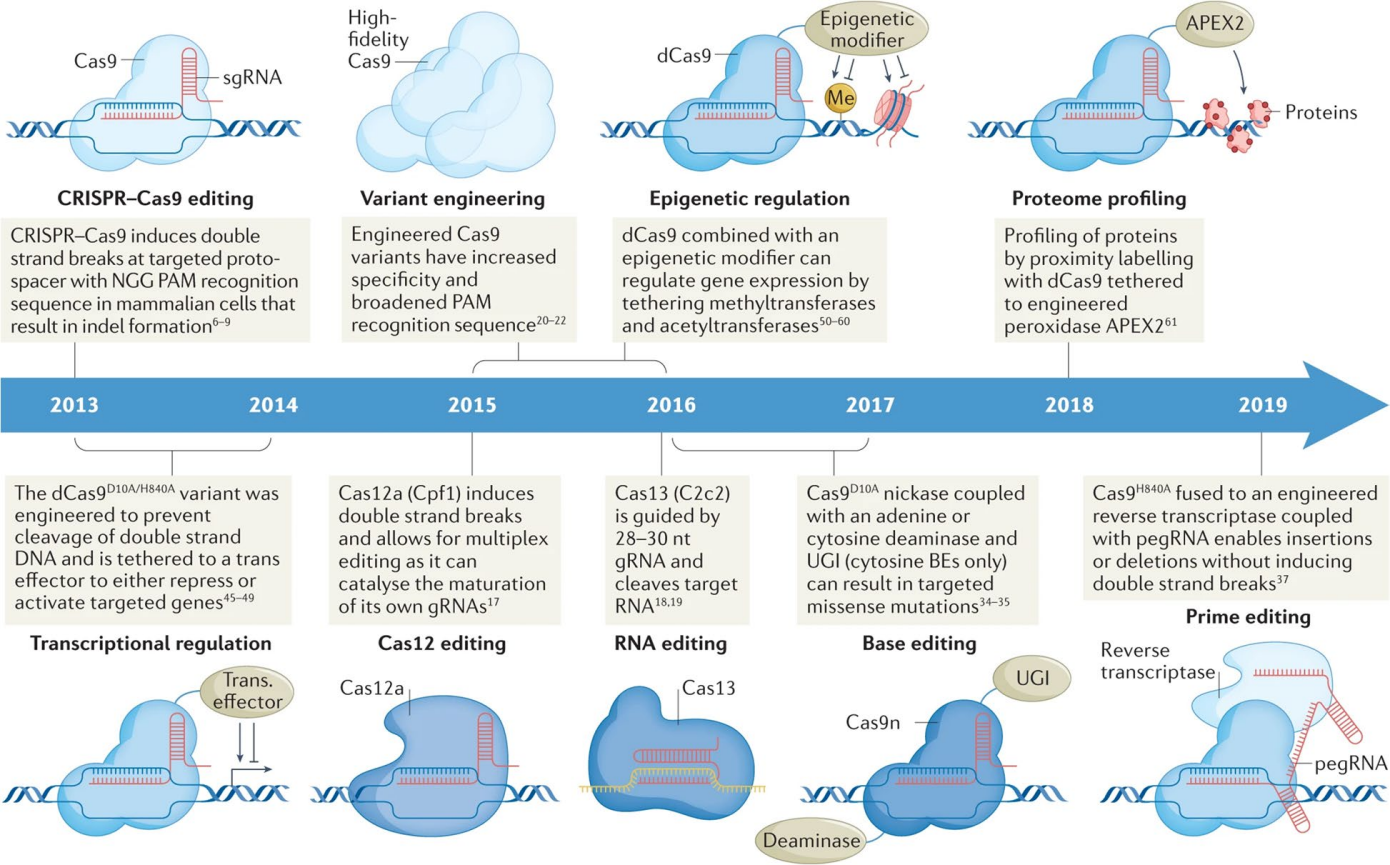
The evolution of CRISPR tools that have been harnessed in the investigation of cancer biology. Since the inception of CRISPR-associated 9 (Cas9) gene editing in mammalian cells, there has been a rapid expansion in the field of CRISPR technology. This expansion has led to the development of various specialized CRISPR variants designed to tackle specific challenges. Scientists have created these variants through deliberate design and evolutionary processes, resulting in improved flexibility in recognizing protospacer adjacent motifs (PAMs) and increased precision in target selection. Additionally, they've harnessed naturally occurring variants from different bacterial species, like Cas12a (Cpf1) and Cas13, for effective combinatorial knockout (KO) and precise RNA targeting, respectively. To broaden the range of CRISPR applications, researchers have combined transcriptional effectors with catalytically inactive Cas9 (dCas9), allowing precise targeting of the transcriptome and epigenome. Furthermore, CRISPR base editing has enabled the introduction of specific transition mutations using a Cas9 nickase (Cas9n) fused with adenine or cytosine deaminase. In the case of cytosine base editing enzymes (BEs), they use a uracil glycosylase inhibitor (UGI) to prevent base excision repair and promote C > T transition mutations. A significant advancement known as prime editing has emerged, which involves fusing a dCas9 with a reverse transcriptase, enabling the engineering of various mutation types, such as missense mutations, insertions, and deletions. This is guided by a sequence template and an extended prime editing guide RNA (pegRNA). Additionally, to facilitate unbiased proteome mapping, researchers have employed engineered ascorbate peroxidase (APEX2) tethered to dCas9, enabling targeted biotinylation at specific genomic locations. Reprinted from [11] with permission from Springer Nature

The use of CRISPR (Clustered Regularly Interspaced Short Palindromic Repeats) in cancer therapy has the potential to revolutionize the way for treating different diseases [1]. CRISPR technology allows for precise and efficient manipulation of the genome, and its application in cancer research has the potential to target specific genetic mutations that drive the growth and spread of tumors [2]. In recent years, there has been a growing body of research exploring the use of CRISPR-based gene editing in cancer therapy, with several preclinical studies and clinical trials demonstrating promising results [3]. The discovery of CRISPR technology in 2012 marked a significant milestone in the field of genome editing [4]. Figure 1 illustrates the evolution of CRISPR tools used for exploring cancer biology. CRISPR-associated enzymes, such as Cas9, can be programmed to target specific DNA sequences, and when combined with guide RNAs, can be used to cut, modify or delete genes in a precise manner [4]. This technology has been used in a wide range of applications, including basic research, gene therapy, and agriculture [1]. However, its potential application in cancer research has attracted particular interest due to the ability to target the genetic mutations that drive the growth and spread of tumors [3]. There are several different CRISPR-based strategies that have been proposed for cancer therapy [4]. One approach is to inactivate genes that drive tumor growth. For example, using CRISPR to inactivate the oncogene MYC has been proposed as a way to halt tumor growth. The MYC gene is known to be overactive in many types of cancer, and its inactivation could potentially slow down or stop the progression of the disease [5]. Another approach is to enhance the immune response to cancer cells. For example, researchers have used CRISPR-based gene editing to knockout or decrease the expression of the PD-1 protein on T cells, which helps to improve their ability to target and kill cancer cells [6]. Additionally, CRISPR-based gene editing can be used to repair genetic mutations that cause cancer, such as in the case of inherited forms of cancer caused by BRCA1 and BRCA2 mutations [7]. For example, studies have shown that CRISPR-Cas9 can be utilized to correct BRCA1 mutations in human cells, demonstrating the potential for this technology in cancer therapy [8]. Furthermore, CRISPR-based gene editing can also be employed in immunotherapeutic strategies for cancer treatment. For instance, T cells can be engineered using CRISPR to express receptors that specifically target tumor cells, enhancing the body's immune response against cancer [9]. Preclinical studies and clinical trials have been conducted using these strategies, and they have demonstrated promising results [3]. For example, inactivating the MYC oncogene in animal models of lymphoma has been shown to reduce tumor growth. Similarly, increasing the expression of PD-1 on T cells has been shown to enhance the ability of these cells to target and kill cancer cells in animal models [1]. However, despite the promising results obtained in preclinical studies, there are still many challenges that need to be overcome for CRISPR-based cancer therapy to become a viable clinical option [4]. One of the main challenges is the risk of non-specific site effects, which can occur when CRISPR enzymes target unintended regions of the genome. Safety and delivery are also critical challenges that need to be addressed [10].

In this review article, we will provide an overview of the current state of the field of CRISPR-based gene editing in cancer therapy, highlighting the most promising results and the challenges that still need to be overcome. We will describe the different CRISPR-based strategies that have been proposed for cancer therapy, summarize the current state of preclinical studies and clinical trials, and discuss the challenges and limitations that need to be overcome for CRISPR-based cancer therapy to become a viable clinical option. We will also provide an overview of future directions for research, development and discuss the potential implications of CRISPR-based cancer therapy for the future of cancer treatment and healthcare.

## CRISPR-based strategies for cancer therapy

**Fig. 2:**
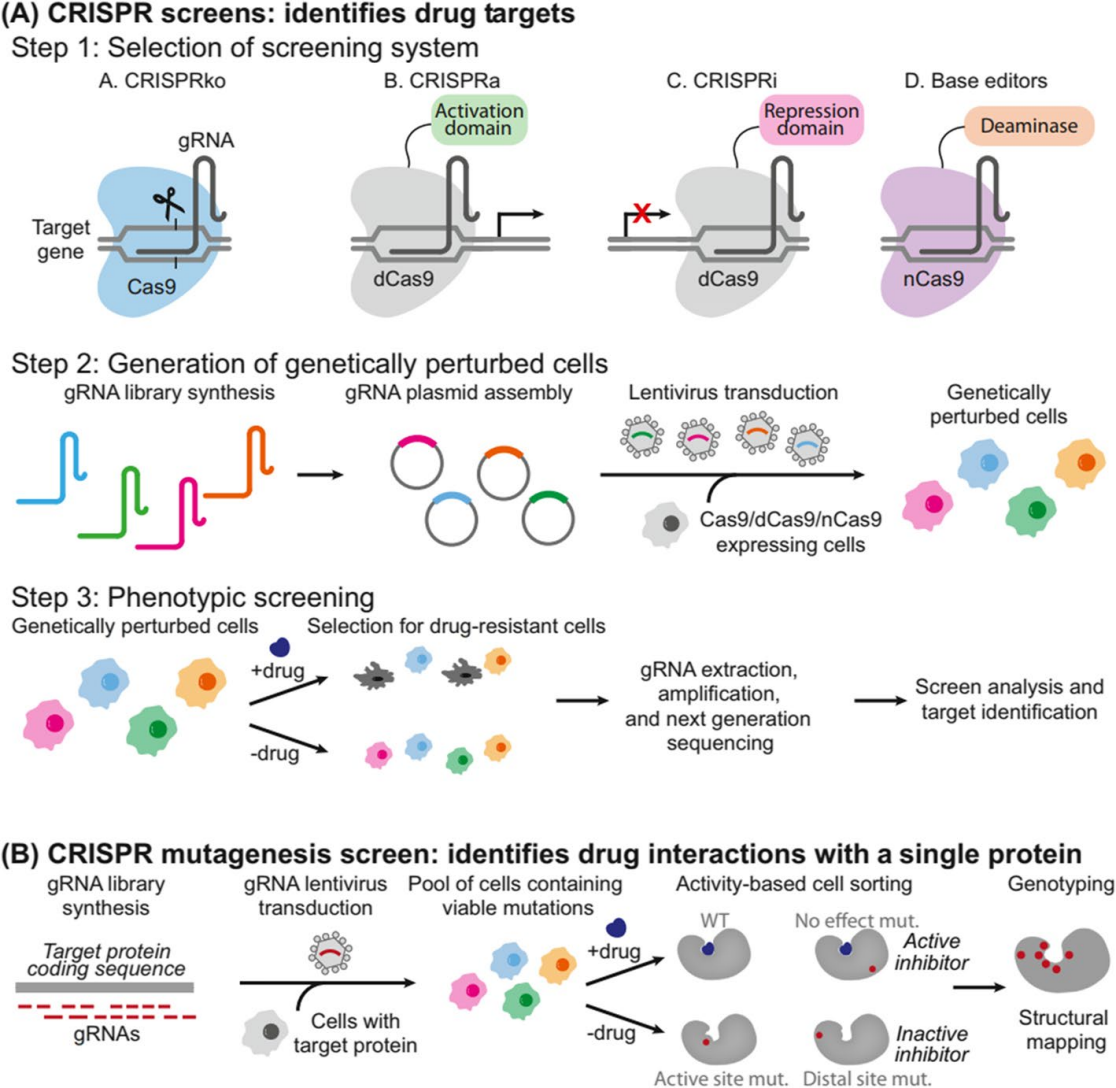
Different workflows used in CRISPR screening and mutagenesis. The CRISPR screening procedures commence by selecting the appropriate screening system, offering various options: **A** CRISPRko, where Cas9 is employed to disrupt genes, resulting in the generation of premature stop codons or frameshift mutations; CRISPRa, involving the attachment of activation domains (e.g., VPR, VP64) to dCas9, resulting in enhanced transcription of target genes; CRISPRi, on the contrary, employs repression domains (e.g., KRAB) tethered to dCas9, leading to a reduction in the transcription of target genes; Base editing screen, which uses a base editor (e.g., cytosine deaminase or adenine deaminase) with or without a uracil DNA glycosylase inhibitor to induce mutations without causing double-strand breaks. Once the suitable CRISPR screening method is chosen, the gRNA library is introduced into cells, creating a genetically altered cell population. These cells are exposed to drugs to select for drug-resistant populations. Subsequently, the gRNAs are extracted from the cells, amplified via PCR, and their target genes are determined using next-generation sequencing. **B** On the other hand, CRISPR mutagenesis screening begins with a gRNA library designed to induce in-frame mutations in the target protein coding sequence. After transducing the cells with the gRNA library, viable cells with protein variants are subjected to drug treatment, both with and without the drug. Activity-based cell sorting is used to enrich cells carrying mutations that make the drug ineffective, thereby identifying drug-resistant cells. Finally, the enriched cells are genotyped using deep sequencing to analyze structural changes and detect any escape mutants. Reprinted from [14] with permission from Cell Press

**Fig. 3:**
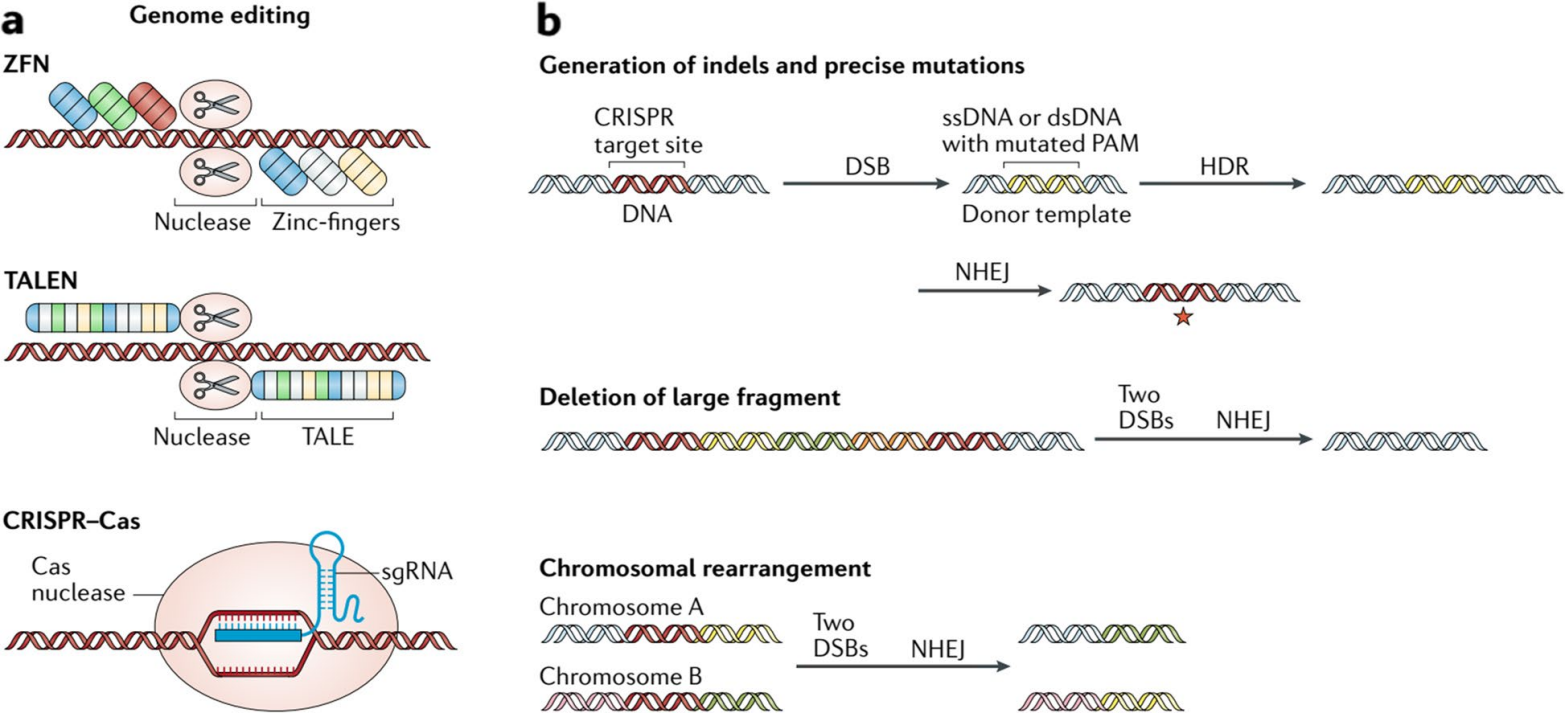
The various mechanisms employed for gene editing. In the first part (**a**), Zinc-finger nucleases (ZFNs), transcription activator-like effector nucleases (TALENs), and CRISPR-Cas systems are capable of generating double-strand breaks (DSBs) at precise locations within the genome. Moving on to the second part (**b**), the introduction of DNA sequences or mutations into the DNA can be achieved by means of homology-directed repair (HDR) or non-homologous end joining (NHEJ) processes with the aid of a donor template. In mammalian cells, CRISPR-induced DSBs are generally mended via NHEJ, which can result in the incorporation of small insertions and/or deletions (indels), leading to gene inactivation due to frameshift mutations. When two DSBs occur on the same chromosome, a substantial segment can be deleted, whereas DSBs on different chromosomes can give rise to chromosomal rearrangements. The abbreviations found in the figure include dsDNA (double-stranded DNA), PAM (protospacer adjacent motif), sgRNA (single-guide RNA), ssDNA (single-stranded DNA), and TALE (transcription activator-like effector). Reprinted from [15] with permission from Springer Nature

CRISPR-based gene editing technology has the potential to revolutionize the way for treating cancer by allowing for precise and efficient manipulation of the genome to target specific genetic mutations that drive the growth and spread of tumors [12]. Figure 2 highlights the step-by-step process of CRISPR screening, starting with the identification of specific gene targets. Subsequently, it illustrates the design and construction of CRISPR guide RNA libraries, essential for precise genomic targeting. Following this, the delivery of CRISPR components into the target cells is depicted, demonstrating the methods employed for gene editing in a wide range of cell types. The next stage outlines the application of selective pressures to identify cells with desired genetic alterations, and ultimately, the evaluation of the screening results. Figure 3 illustrates the various mechanisms of gene editing. Several different CRISPR-based strategies have been proposed for cancer therapy, each with their own advantages and limitations [13]. Table 1 outlines several CRISPR-based strategies for cancer therapy.

**Table 1:** CRISPR-based strategies for cancer therapy

Strategy	Mechanism of Action	Advantages	Disadvantages	Preclinical/clinical results	Ref
Inactivating genes that drive tumor growth	Targeting and disrupting oncogenes and tumor suppressor genes to stop cancer cell growth and induce apoptosis	Precision targeting: CRISPR can be designed to target specific genes or mutations that drive tumor growth, increasing specificity and reducing off-target effects. High efficacy: Studies have shown that inactivating certain genes using CRISPR can result in tumor regression and increased survival in animal models	Potential for off-target effects: While CRISPR offers high specificity, there is still the potential for unintended changes in the genome that could cause harm to the patient. Difficulty targeting specific genes or delivering therapy to tumor site: Some tumors may be difficult to target using current delivery methods	Study showed efficacy of using CRISPR to inactivate KRAS oncogene in mouse model of lung cancer. This is a major milestone since KRAS mutations are notoriously difficult to target with other therapies	[16]
Enhancing immune response to cancer cells	Editing immune cells to recognize and destroy cancer cells, such as by editing T cells to express chimeric antigen receptors (CARs)	Enhances body's natural immune response: By editing immune cells to recognize and attack cancer cells, CRISPR-based immunotherapy can activate the body's natural immune response to fight the cancer. Avoids toxic effects of chemotherapy: Unlike chemotherapy, which can have significant side effects, immunotherapy using CRISPR-edited T cells has the potential to be a more targeted and less toxic treatment approach	Potential for toxicity or immune rejection: There is a risk that CRISPR-edited immune cells could attack healthy cells or be rejected by the patient's immune system. Limited availability of specific T cells for editing: It can be challenging to obtain a sufficient number of T cells for editing, which could limit the widespread use of this approach	Study reported complete remission in two out of three patients treated with CRISPR-edited T cells in a trial for refractory lymphomas. This is a promising result, although more research is needed to determine the safety and efficacy of this approach in larger patient populations	[17]
Repairing genetic mutations that cause cancer	Correcting genetic mutations in tumor suppressor genes, DNA repair genes, or other driver genes	Precision targeting: CRISPR can be used to correct specific genetic mutations that cause cancer, potentially leading to long-term benefits. Potential for long-term benefits: Repairing genetic mutations that cause cancer could potentially result in long-term benefits for patients	Potential for off-target effects: As with other CRISPR-based approaches, there is a risk of unintended changes to the genome that could cause harm to the patient. Difficulty delivering therapy to tumor site: It can be challenging to deliver CRISPR-based therapy directly to the tumor site	Promising results in preclinical studies, such as using CRISPR to correct BRCA1 mutations in ovarian cancer cells	[18]
Delivering cancer-killing molecules directly to tumor cells	Using CRISPR to edit the genome of a virus or bacteria to specifically target cancer cells, delivering therapeutic molecules such as toxins or immune modulators directly to tumor cells	Precision targeting: By editing the genome of a virus or bacteria to specifically target cancer cells, CRISPR-based therapy can offer highly targeted and specific delivery of therapeutic molecules to tumor cells. High efficacy: Studies have shown that CRISPR-mediated delivery of cancer-killing molecules can result in tumor regression and increased survival in animal models	Potential for off-target effects: As with other CRISPR-based approaches, there is a risk of unintended changes to the genome that could cause harm to the patient. Limited availability of specific viruses or bacteria for editing: It can be challenging to obtain a sufficient number of specific viruses or bacteria for editing, which could limit the widespread use of this approach	Promising results in preclinical studies, such as using CRISPR to deliver CRISPRa to activate tumor-suppressive microRNAs in liver cancer cells. Other examples include using CRISPR to deliver toxic payloads to tumor cells, such as in a study where CRISPR was used to engineer bacteria to produce a toxin that specifically targets cancer cells in a mouse model of pancreatic cancer	[17, 18]

### Inactivation of oncogenes

The mechanism of CRISPR-based strategies in inactivating oncogenes begins with the identification of specific oncogenes that play critical roles in cancer development [19]. Oncogenes are often associated with mutations or abnormal gene amplifications that result in the overexpression of their respective proteins, leading to uncontrolled cell growth and proliferation [20]. Once the target oncogene has been identified, researchers design a gRNA that specifically recognizes and binds to the mutated or amplified region of the oncogene [19]. One approach is to inactivate genes that drive tumor growth. For example, inactivating the MYC oncogene has been shown to reduce tumor growth in animal models of lymphoma [21]. This strategy is based on the principle that cancer cells have genetic mutations that lead to the over-expression of oncogenes, which promote cell growth and proliferation. Inactivating these oncogenes can stop the growth of cancer cells [22]. CRISPR-based approaches can be seamlessly integrated with other cancer therapies to maximize efficacy and improve treatment outcomes [23]. For instance, combining CRISPR with chemotherapy allows for the precise editing of genes involved in drug resistance, sensitizing cancer cells to chemotherapeutic agents [24]. Additionally, CRISPR can be used to engineer patient-derived immune cells, such as T cells, to express CARs that enhance their tumor-targeting capabilities in combination with CAR-T cell therapy [22]. Furthermore, by disrupting immune checkpoint genes in cancer cells, CRISPR augments the effectiveness of immunotherapies like immune checkpoint inhibitors [25]. Another example is integrating CRISPR with targeted therapies, where simultaneous targeting of multiple critical pathways using gene editing can overcome resistance and potentiate the effects of targeted drugs [26]. By employing CRISPR to enhance drug delivery, researchers can modify tumor cells or the tumor microenvironment to improve the penetration of therapeutics, thereby augmenting the impact of various cancer treatments. These examples demonstrate the versatility of CRISPR in synergizing with other cancer therapies and pave the way for more effective and personalized treatment approaches in the fight against cancer [27].

### Enhancement of immune response

**Fig. 4:**
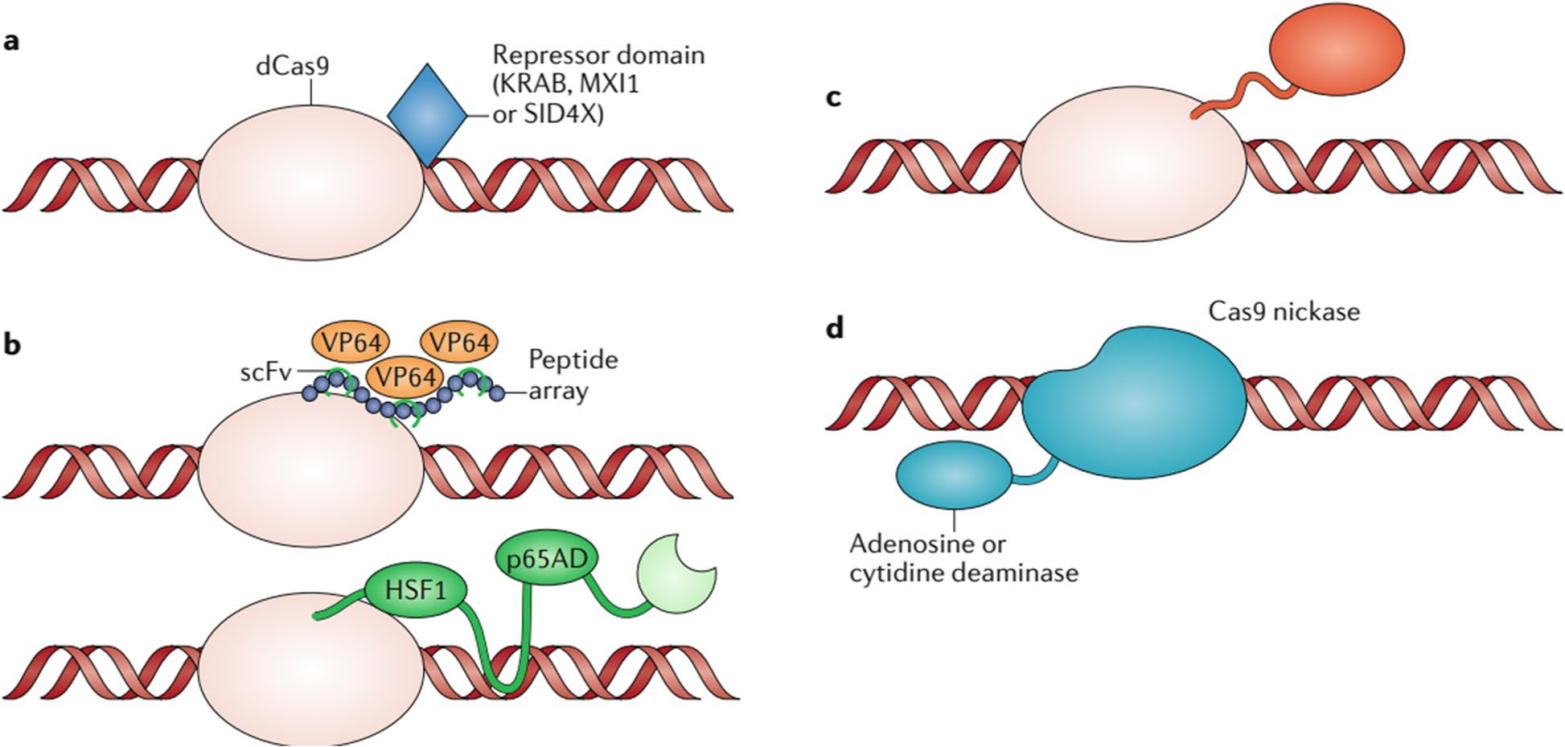
The diverse applications of CRISPR technology within cancer research. In section **a**, the paragraph explains that the inhibition of a specific gene can be accomplished by combining Deactivated Cas9 (dCas9) with repressor domains. In section **b**, it discusses how the fusion of dCas9 with activation domains can stimulate the expression of a particular gene. Furthermore, it mentions that augmenting the binding of additional transcription activators to a single-guide RNA or dCas9 can intensify the expression of target exons. In section **c**, it states that when dCas9 is fused with epigenetic regulators, it can either repress or activate transcription. In section **d**, the paragraph talks about the focused introduction of point mutations in the genome, which is made possible by combining dCas9 with adenosine deaminase or cytidine deaminase, allowing for precise genetic modifications. Additionally, it provides explanations for the abbreviations KRAB (Kruppel-associated box) and scFv (single-chain variable fragment). Reprinted from [15] with permission from Springer Nature

The mechanism revolves around harnessing the potential of the CRISPR-Cas system, a natural defense mechanism found in bacteria and archaea, which has been adapted for targeted gene editing in various organisms [28]. To enhance the immune response, scientists utilize CRISPR-Cas to edit specific genes involved in immune regulation and response pathways [29]. Gene editing can be employed to knockout genes that negatively regulate the immune system, thus bolstering its activity [28]. Additionally, CRISPR-based techniques enable the precise insertion of beneficial genes, such as cytokines or other immune mediators, to enhance the immune response against particular antigens [23]. Furthermore, CRISPR-Cas can be utilized to engineer immune cells like T-cells and NK cells, improving their functionality and specificity towards cancer cells or infected targets [30]. Moreover, CRISPR-Cas enables the development of genetic vaccines, where specific antigen-encoding genes are delivered into host cells to elicit a robust and targeted immune response. These breakthroughs in CRISPR-based immune enhancement hold great promise for combating infectious diseases, cancer, and other conditions where bolstering the immune system is critical for effective treatment [31]. Researchers have used CRISPR-based gene editing to increase the expression of the PD-1 protein on T cells, which helps to improve their ability to target and kill cancer cells [32]. CRISPR-Cas enhances the immune response by enabling precise gene editing [33]. Scientists can target specific genes involved in immune regulation and response pathways. By knocking out genes that negatively regulate the immune system, CRISPR-Cas increases the overall activity of the immune system [23]. Additionally, beneficial genes, such as cytokines or other immune mediators, can be inserted using CRISPR-Cas to further enhance the immune response against specific antigens [34]. CRISPR-based gene editing can sometimes result in non-specific site effects, where unintended changes occur in other parts of the genome. These non-selective site effects may lead to unwanted alterations in gene function and could pose safety concerns in the context of immune enhancement [35, 36]. It is essential to thoroughly evaluate and minimize these undesirable site effects to ensure the safety and effectiveness of CRISPR-based strategies [28]. CRISPR-Cas can be utilized to modify immune cells, such as T-cells and NK cells, to improve their functionality and specificity in targeting cancer cells or infected cells. By editing the genes responsible for cell receptors and signaling pathways, researchers can enhance the ability of immune cells to recognize and destroy specific targets [33]. Understanding the mechanisms and optimizing the protocols for this gene editing process is crucial for developing successful immune cell-based therapies [28]. While CRISPR-based strategies show great promise in enhancing the immune response, it is essential to investigate their long-term effects on the host's immune system [30]. Prolonged activation or manipulation of immune pathways could potentially lead to immune system dysregulation, autoimmunity, or immune exhaustion [37]. Understanding the impact of CRISPR-based immune enhancement on the overall immune function and homeostasis is crucial for safe and sustainable clinical applications. Long-term follow-up studies in animal models and clinical trials will be necessary to address these concerns [23]. Figure 4 illustrates various applications of CRISPR in cancer research.

### Repair of genetic mutations

CRISPR-based gene editing can also be used to repair genetic mutations that cause cancer, such as in the case of inherited forms of cancer caused by BRCA1 and BRCA2 mutations [37]. The CRISPR-Cas9 system is highly specific in targeting genetic mutations due to the guide RNA's ability to recognize and bind to a particular DNA sequence [38]. However, undesirable site effects can occur, where the Cas9 enzyme might inadvertently cleave similar sequences elsewhere in the genome [39]. Continuous advancements in bioinformatics and experimental techniques are improving the specificity and reducing non-selective site effects, making it crucial to evaluate the system's precision in repairing genetic mutations [37]. Assessing the efficiency and accuracy of CRISPR-mediated repair methods, such as HDR and NHEJ, is vital. HDR can accurately introduce the desired genetic changes by utilizing a donor template, but its efficiency is often lower compared to NHEJ, which can result in insertions or deletions without a template [40]. Understanding the balance between efficiency and accuracy will help optimize the choice of repair mechanism for specific genetic mutations [41]. While CRISPR has shown great promise, there might be unforeseen consequences of manipulating the genome. These could include non-selective site mutations or large-scale genomic rearrangements, which may introduce new genetic abnormalities or cause unintended effects on gene regulation [42]. Careful evaluation and thorough assessment of potential unintended outcomes are essential to ensure the safety and reliability of CRISPR-based strategies [37]. Understanding the stability of CRISPR-induced genetic repairs is critical for assessing the long-term viability of potential treatments [40]. Genetic modifications must be stable and faithfully passed on during cell divisions to provide lasting therapeutic benefits. Investigating the heritability and stability of repaired genetic mutations will shed light on the longevity and efficacy of CRISPR-based strategies [41]. When using CRISPR-Cas9 for in vivo applications, it is crucial to evaluate potential immune responses to the Cas9 protein and guide RNA [37]. The immune system might recognize these components as foreign entities, leading to unwanted immune reactions or clearance of CRISPR-modified cells. Understanding the immunogenicity of CRISPR components will aid in developing strategies to minimize immune responses and enhance the safety and success of gene therapies [41].

### Delivery of cancer-killing molecules

CRISPR-based strategies have revolutionized cancer treatment by enabling the precise delivery of cancer-killing molecules to targeted cells [43]. The mechanism behind this innovative approach involves utilizing the CRISPR-Cas system, a powerful gene-editing tool, to effectively locate and destroy cancerous cells while sparing healthy ones [44]. Firstly, researchers design guide RNA molecules that specifically target and bind to cancer cell DNA, serving as molecular homing devices. Secondly, these guide RNAs are loaded onto a CRISPR-associated protein (Cas) complex, forming the CRISPR-Cas ribonucleoprotein (RNP) complex. This RNP complex can be seen as a delivery system for cancer-killing molecules, which is a crucial part of CRISPR/Cas9-based cancer gene therapy, where gene-editing technology is leveraged to treat cancer by editing the genetic material within cancer cells [45]. Thirdly, the RNP complex, along with cancer-killing molecules, is then introduced into the patient's body, either through direct injection or as part of engineered immune cells, such as T-cells. Various delivery systems, such as nanotechnology-based delivery systems, have been explored to ensure the efficient delivery of the CRISPR-Cas system and cancer-killing molecules to target cells [46]. Fourthly, once inside the cancer cells, the CRISPR-Cas RNP complex precisely cuts and deactivates the oncogenes responsible for the malignancy, while the delivered cancer-killing molecules initiate apoptosis (cell death) or render the cancer cells susceptible to the body's immune response [47]. The ultimate goal is to optimize the delivery and application of the CRISPR–Cas system for clinical cancer therapy, overcoming challenges associated with in vivo delivery, to ensure the safety and effectiveness of this therapeutic approach [48]. Finally, the targeted destruction of cancer cells occurs, leading to tumor regression while minimizing damage to healthy tissues. This breakthrough mechanism holds immense promise in the development of highly specific and efficient cancer therapies, potentially revolutionizing the landscape of oncology treatments in the future [49]. Guide RNA molecules are designed to have complementary sequences that specifically bind to the DNA of cancer cells. This specificity is achieved by identifying unique genetic markers or mutations present in cancer cells but not in healthy cells. By targeting these specific sequences, guide RNAs can effectively distinguish cancerous cells from healthy ones [50]. The Cas complex serves as a carrier for the guide RNA molecules. It forms a complex with the guide RNA, creating the CRISPR-Cas RNP complex [51]. The Cas protein provides the necessary machinery to recognize the guide RNA and facilitates its binding to the target DNA within cancer cells. This complex acts as a powerful molecular scissor, cutting and deactivating the oncogenes responsible for cancer growth [50]. The CRISPR-Cas RNP complex can be introduced into the patient's body through different methods. One approach involves direct injection into the target tissue or tumor site [49]. Another method involves engineering immune cells, such as T-cells, to express the CRISPR-Cas RNP complex. These engineered immune cells can then be reintroduced into the patient's bloodstream, where they can specifically target and attack cancer cells [51]. Once inside the cancer cells, the CRISPR-Cas RNP complex locates the targeted DNA sequences and precisely cuts them, deactivating the oncogenes responsible for the malignancy. This deactivation leads to either the initiation of apoptosis (cell death) in cancer cells or renders them more susceptible to the body's immune response, resulting in their destruction [49]. The guide RNA molecules are designed to specifically target cancer cells by binding to unique genetic markers or mutations found in those cells. By selectively targeting cancerous cells, the CRISPR-Cas RNP complex effectively spares healthy cells from damage, minimizing potential side effects [52]. Additionally, the use of engineered immune cells allows for even greater specificity in targeting cancer cells, further reducing the impact on healthy tissues [52, 53].

## Preclinical studies and clinical trials for CRISPR-based cancer therapy

**Fig. 5:**
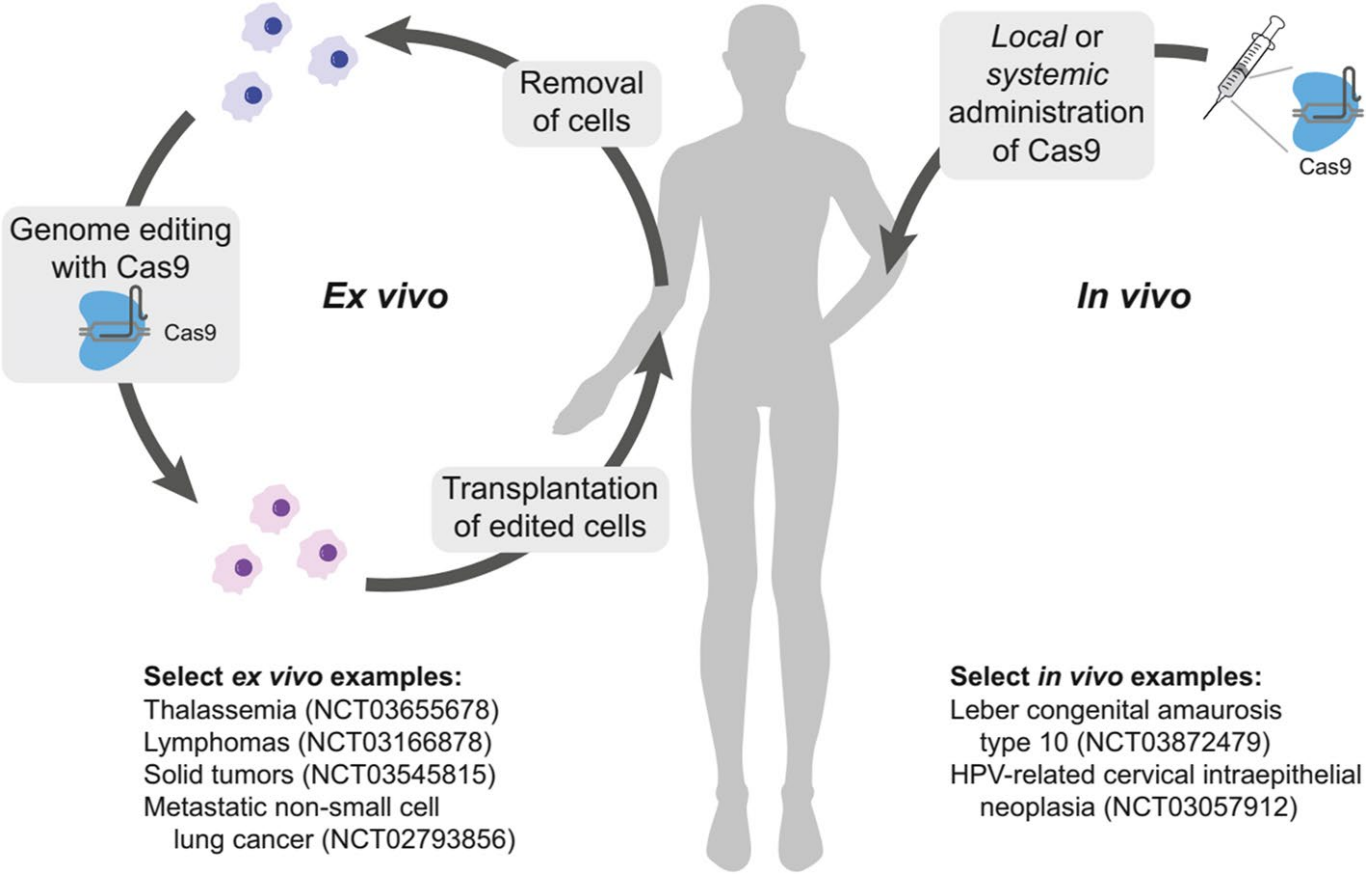
Different strategies for editing cells using CRISPR technology in patients. On the left, ex vivo applications involve first isolating cells, then expanding and editing them before transplanting them back. On the right, in vivo editing occurs by administering CRISPR-Cas9 (or dCas9, not shown) locally or systemically using viral packaging or nanoparticles. The figure also highlights specific clinical trials. Abbreviations used include CRISPR (clustered regularly interspaced short palindromic repeats), dCas9 (dead Cas9), and HPV (human papillomavirus). Reprinted from [14] with permission from Cell Press

Although there have been significant advancements in the CRISPR gene-editing technology, with over 800 cell and gene therapy programs in existence, only a limited number of CRISPR-based tools have successfully advanced beyond preclinical trials [51]. Other gene editing methods, such as TALENs and ZFNs, have been explored extensively in clinical settings and have been reviewed elsewhere. Figure 5 illustrates different strategies for editing cells using CRISPR technology in patients. The development of CRISPR-based cancer therapy is a rapidly evolving field that is moving from preclinical studies to clinical trials [54]. Preclinical studies are essential for evaluating the safety and efficacy of CRISPR-based cancer therapy before it can be tested in humans [55]. Clinical trials are the final step in the development process and are used to determine the safety and efficacy of a therapy in humans.

### Preclinical studies

**Fig. 6:**
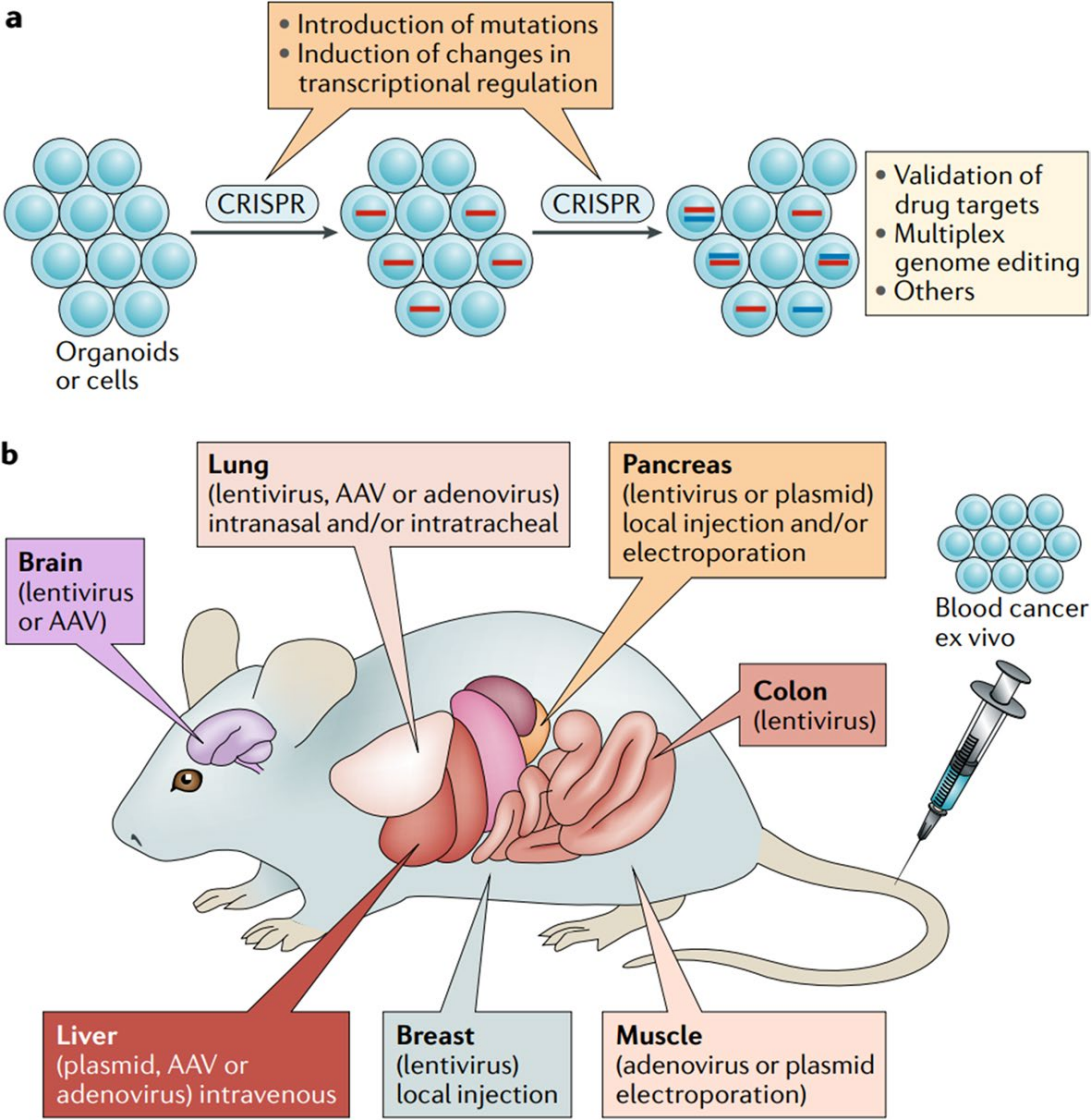
Employing CRISPR for creating cancer models in cells and mice. In the initial case (**a**), cultured cells or organoids undergo genome editing through CRISPR, which facilitates the incorporation of alterations or adjustments in transcriptional control at one or more phases. In the latter case (**b**), CRISPR mechanisms can be transferred to animal models using diverse methods, thereby enabling them to target numerous tissues and organs. One such approach involves utilizing adeno-associated viruses (AAV) for delivery. Reprinted from [15] with permission from Springer Nature

Preclinical studies are a critical step in the development of any new cancer therapy, including those based on CRISPR technology [56]. Several studies are conducted in laboratory animals, such as mice and rats, and are used to evaluate the safety, efficacy, and potential side effects of a new therapy. Figure 6 illustrates the application of CRISPR in cancer modeling for cells and mice. Martinez-Lage et al. presented a clever preclinical approach targeting oncogenic gene fusions, aiming for both tumor cell selectivity and disruption of a tumor-promoting genetic lesion. This strategy took advantage of the unique fusion characteristic and demonstrated potential effectiveness [57]. Another preclinical example by Gao et al. focused on exploiting nuclear factor-κB (NF-κB), which is selectively activated in cancer cells, to drive the transcription of CRISPR-Cas13a components. This resulted in cancer cell-restricted oncogene silencing, offering a promising avenue for cancer therapy [58]. Table 2 presents a summary of preclinical studies exploring the potential of CRISPR-based cancer therapy across various cell types.

**Table 2:** Preclinical studies of CRISPR-based cancer therapy

Cell/Tissue Type	CRISPR Approach	Results	Limitations/Challenges	Ref
Mouse and Human T cells	Knockout of PD-1	Enhanced T cell activity against cancer cells, prolonged survival in mice	Safety concerns with long-term PD-1 knockout	[59]
Ovarian Cancer Cells	Knockout of oncogene	Reduced cell proliferation and colony formation	Limited assessment of off-target effects	[60]
Lung Cancer Cells	Knockout of mutant EGFR	Reduced cell viability and tumor growth in mice	Off-target effects in some cells	[61]
Triple-Negative Breast Cancer Cells	Knockout of AXL	Reduced tumor growth and increased sensitivity to chemotherapy	Limited assessment of off-target effects	[62]
Various Cancer Cell Lines	Knockout of HIF-1α	Reduced tumor growth and increased sensitivity to radiation therapy	Limited assessment of off-target effects	[63]
Melanoma Cells	Knockout of BRAF V600E	Reduced tumor growth and increased sensitivity to targeted therapy	Off-target effects in some cells	[64]
Leukemia Cells	Knockout of MCL-1	Induced apoptosis and reduced tumor growth	Limited assessment of off-target effects	[65]
Human T cells	Knockout and overexpression of NKG2D	Enhanced tumor cell recognition and killing by CAR-T cells	Safety concerns with long-term NKG2D overexpression	[66, 67]
Pediatric Solid Tumors	Knockout of fusion oncogenes	Reduced tumor growth and increased sensitivity to chemotherapy	Limited assessment of off-target effects	[68]
Prostate Cancer Cells	Knockout of androgen receptor	Reduced cell proliferation and tumor growth in mice	Off-target effects in some cells	[69]
Pancreatic Cancer Cells	Knockout of TGF-β pathway genes	Reduced tumor growth and increased sensitivity to chemotherapy	Limited assessment of off-target effects	[70]
Breast Cancer Cells	Knockout of PAK1 C-terminus	Reduced cell proliferation and tumor growth in mice	Off-target effects in some cells	[71]
Diffuse Large B-cell Lymphoma Cells	Knockout of BCL6	Induced apoptosis and reduced tumor growth	Limited assessment of off-target effects	[72]
Glioblastoma Cells	Knockout of TERT promoter mutations	Reduced cell proliferation and tumor growth in mice	Limited assessment of off-target effects	[73]
Acute Myeloid Leukemia Cells	Knockout of GATA2	Induced differentiation and reduced tumor growth	Limited assessment of off-target effects	[74]
Human Hematopoietic Stem Cells	Knockout of BCL11A enhancer	Induced fetal hemoglobin expression and reduced sickle cell symptoms	Limited assessment of off-target effects	[75]
B Cell Acute Lymphoblastic Leukemia Cells	Knockout of CD19	Induced apoptosis and reduced tumor growth in mice	Off-target effects in some cells	[76]
Diffuse Large B-cell Lymphoma Cells	Knockout of EZH2	Reduced cell proliferation and tumor growth in mice	Limited assessment of off-target effects	[77]
Glioma Cells	Knockout of IDH1	Reduced cell viability and tumor growth in mice	Off-target effects in some cells	[78]
Neuroblastoma Cells	Knockout of MYCN	Induced apoptosis and reduced tumor growth	Limited assessment of off-target effects	[79]
Ovarian Cancer Cells	Knockout of MUC16	Reduced cell proliferation and invasion	Limited assessment of off-target effects	[80]
Alveolar Rhabdomyosarcoma Cells	Knockout of PAX7-FOXO1	Reduced cell proliferation and tumor growth in mice	Off-target effects in some cells	[81]
Esophageal Cancer Cells	Knockout of SOX2	Reduced cell proliferation and colony formation	Limited assessment of off-target effects	[82]
Hepatocellular Carcinoma Cells	Knockout of TERT	Reduced cell proliferation and tumor growth in mice	Limited assessment of off-target effects	[83]
Colorectal Cancer Cells	Knockout of Wnt/β-catenin pathway genes	Reduced cell proliferation and tumor growth in mice	Off-target effects in some cells	[84]
Breast Cancer Cells	Knockout of P53	Increased cell proliferation and colony formation	Off-target effects in some cells	[85]
Pancreatic Cancer Cells	Knockout of KRAS	Reduced cell viability and tumor growth in mice	Limited assessment of off-target effects	[86]
Cervical Cancer Cells	Knockout of BIRC5	Reduced cell proliferation and tumor growth in mice	Limited assessment of off-target effects	[87]
Prostate Cancer Cells	Knockout of EZH2	Reduced cell proliferation and colony formation	Off-target effects in some cells	[79]
Osteosarcoma Cells	Knockout of HIF-1α	Reduced cell proliferation and tumor growth in mice	Limited assessment of off-target effects	[88]
Acute Lymphoblastic Leukemia Cells	Knockout of MYB	Induced apoptosis and reduced tumor growth	Limited assessment of off-target effects	[89]
Renal Cell Carcinoma Cells	Knockout of HIF-2α	Reduced cell proliferation and tumor growth in mice	Limited assessment of off-target effects	[90]
Hepatocellular Carcinoma Cells	Knockout of SALL4	Reduced cell proliferation and tumor growth in mice	Limited assessment of off-target effects	[91]
Melanoma Cells	Knockout of CDK6	Reduced cell proliferation and tumor growth in mice	Limited assessment of off-target effects	[92]
Acute Myeloid Leukemia Cells	Knockout of ASXL1	Induced differentiation and reduced tumor growth	Limited assessment of off-target effects	[93]

P53 is a critical tumor suppressor protein responsible for regulating cell cycle progression and preventing the formation of cancerous cells [94]. In the context of CRISPR/Cas9 genome editing, the activity of P53 becomes a crucial concern as the use of this technology may lead to non-targeted site effects, causing unintended DNA damage [95]. Therefore, it is essential to evaluate the impact of CRISPR/Cas9 on P53 expression and function to ensure the safety and efficacy of the editing process [96]. P53 plays a crucial role in monitoring the integrity of the cell's DNA and inducing cell cycle arrest or apoptosis in case of DNA damage [97]. Unfortunately, P53 is susceptible to mutations, leading to its inactivation or dysfunction. These mutations are common in many cancer types, including lung cancer, and contribute to uncontrolled cell growth and tumor development [98]. Modifying the P53 gene can restore its function, leading to the suppression of cancer cell growth [94]. When the modified P53 gene is activated, it enhances the production of the P21 protein, a well-known tumor suppressor that regulates the cell cycle. Increased P21 expression induces cell cycle arrest, preventing cancer cells from proliferating uncontrollably [96]. Moreover, the activation of P21 also makes cancer cells more susceptible to chemotherapy, as cells with active P21 proteins are more prone to apoptosis when exposed to chemotherapy drugs. P21, also known as cyclin-dependent kinase inhibitor 1A (CDKN1A), is a cyclin-dependent kinase inhibitor that plays a pivotal role in regulating the cell cycle and promoting cell cycle arrest. In the context of CRISPR/Cas9 genome editing, P21 may act as a double-edged sword [99]. On one hand, its upregulation in response to DNA damage caused by CRISPR/Cas9 may induce cell cycle arrest, preventing cells from proliferating and potentially compromising the effectiveness of the editing process [100]. On the other hand, P21 can promote DNA repair, which might be beneficial for repairing non-specific site effects [101]. Hence, understanding the interplay between P21 and CRISPR/Cas9 is essential for optimizing the editing outcomes [100]. The P21 protein plays a crucial role in controlling the cell cycle by inhibiting CDKs, which are essential for cell division. By inhibiting CDKs, P21 halts the progression of the cell cycle, leading to cell cycle arrest. This pause in cell division allows the cell time to repair DNA damage before continuing with cell replication [94]. Consequently, when P21 is activated, cancer cells are unable to grow and divide rapidly, reducing tumor growth and progression [97]. Cancer cells with active P21 proteins are more responsive to chemotherapy due to their increased susceptibility to apoptosis [100]. Chemotherapy drugs target rapidly dividing cells, and by arresting the cell cycle through P21 activation, the cancer cells become more vulnerable to the cytotoxic effects of these drugs [99]. Additionally, the activation of P21 may also facilitate DNA repair mechanisms, enhancing the cell's ability to detect and repair chemotherapy-induced DNA damage, thus reducing the chance of drug resistance. Dysregulation of P53 or P21 in CRISPR/Cas9 genome editing could lead to several outcomes [101]. Excessive activation of P53 might trigger cell death pathways, resulting in increased toxicity and adverse effects [100]. On the other hand, impaired P53 activity could promote the survival of cells with unintended mutations, potentially leading to tumorigenesis [97]. Similarly, altered expression of P21 could impact the editing efficiency, cell viability, and potential undesirable site effects [100]. Evaluating the consequences of P53 and P21 dysregulation is vital for understanding the safety and reliability of CRISPR/Cas9-based therapies [99]. To minimize P53 and P21-related complications during CRISPR/Cas9 genome editing, optimizing the delivery methods of CRISPR/Cas9 components is crucial [100]. Researchers can explore using advanced delivery systems, such as nanoparticle-based carriers or viral vectors, to improve the efficiency and specificity of targeting [97]. Additionally, employing cell-type-specific promoters for Cas9 expression could reduce non-targeted site effects and limit potential impacts on P53 and P21 expression levels [94]. Moreover, pre-screening potential target sites and rigorously validating guide RNA sequences can aid in selecting the most effective and specific targets, minimizing unintended effects on P53 and P21 pathways [100]. Hartmann et al. (2021) focused on the implementation of CRISPR/Cas9 genome editing to generate murine lung cancer models that accurately represent the mutational landscape of human disease. Lung cancer remains a significant global health issue with low survival rates, highlighting the need for innovative treatments. The researchers aimed to develop surrogate models that mimic the somatic mutations observed in lung cancer patients, as these mutations significantly impact treatment responses. By employing CRISPR-mediated genome editing, the team successfully targeted Trp53 and KRas genes, effectively recreating the classic murine non-small cell lung cancer (NSCLC) model Trp53fl/fl:lsl-KRasG12D/wt. The resulting tumors displayed similar morphology, marker expression, and transcriptional profiles compared to tumors derived from the Trp53fl/fl:lsl-KRasG12D/wt model. The study demonstrated the applicability of CRISPR/Cas9 for in vivo tumor modeling, providing an alternative to conventional genetically engineered mouse models. Interestingly, tumor onset was achieved not only through constitutive Cas9 expression but also by infecting lung epithelial cells of wild-type animals with two distinct adeno-associated viruses (AAVs) encoding different components of the CRISPR machinery. This approach simplified the process by eliminating the need for extensive husbandry to incorporate new genetic features in conventional mouse models. Overall, the utilization of the CRISPR toolbox in cancer research and modeling is rapidly advancing, enabling researchers to efficiently develop new and clinically relevant surrogate models for translational studies [102].

The BRCA1 gene encodes a tumor suppressor protein that plays a crucial role in DNA repair and maintaining genomic stability [103]. When cells experience DNA damage, BRCA1 is involved in signaling pathways that activate P21, a cyclin-dependent kinase inhibitor [104]. P21 inhibits cell cycle progression, allowing time for DNA repair mechanisms to fix the damaged DNA. This activation of P21 helps prevent the propagation of cells with potentially harmful mutations, reducing the risk of tumorigenesis [105]. Mutations in the BRCA1 gene can disrupt its normal function, impairing DNA repair processes and leading to genomic instability [106]. Consequently, the activation of P21 may be compromised, allowing damaged cells to evade cell cycle arrest and repair checkpoints [107]. This increases the likelihood of these cells acquiring additional mutations, potentially leading to the development of cancer [108]. Understanding the intricate interplay between BRCA1 and P21 is crucial for developing targeted therapies and interventions for individuals with BRCA1 mutations or related cancers [109]. The research revealed that modifying the BRCA1 gene resulted in the suppression of cancer cell growth and heightened responsiveness of these cancer cells to chemotherapy [110]. Specifically, the alteration of the BRCA1 gene triggered the activation of the P21 protein, a well-known tumor suppressor protein that contributes to halting the cell cycle [107]. This activation, in turn, caused a reduction in cell growth and made the cancer cells more susceptible to chemotherapy, as P21-activated cells tend to be more responsive to chemotherapy treatment [106, 111]. Some researchers highlight the potential of CRISPR-based base editing as a valuable resource for the functional evaluation and reclassification of variants of uncertain significance (VUSs) in the BRCA1 gene. Furthermore, this investigation tackled the obstacles associated with assessing functionality and determining the pathogenicity of new BRCA1 variants, which are known to substantially elevate the risk of breast and ovarian cancers and are typically identified through clinical genetic testing. To surmount these hurdles, the scientists employed CRISPR-mediated cytosine base editor BE3 for functional analysis. They carried out a comprehensive screening of CRISPR-mediated base editing using 745 guide RNAs targeting all exons in BRCA1, identifying several previously unidentified variants, including c.-97C > T, c.154C > T, c.3847C > T, c.5056C > T, and c.4986 + 5G > A. The study effectively showcased the utility of CRISPR-mediated base editing as a potent instrument for reevaluating variants of uncertain significance (VUSs) in BRCA1, offering valuable insights for clinical management. This reclassification of VUSs in BRCA1 can have substantial implications for patients and healthcare providers. Patients with clarified variant classifications can receive more precise risk assessments and individualized treatment plans, potentially involving heightened surveillance or preventative measures. For healthcare providers, accurate variant classification guarantees appropriate counseling and risk communication for patients and their families [112].

KRAS is a proto-oncogene that, when mutated, plays a crucial role in the development of various cancers, including colon cancer. Mutated KRAS promotes uncontrolled cell growth, leading to tumor formation [113]. Editing the KRAS gene using CRISPR-Cas9 technology can lead to the activation of the P21 protein, a well-known tumor suppressor. P21 promotes cell cycle arrest by inhibiting cyclin-dependent kinases, effectively halting cancer cell growth [114]. CRISPR-Cas9 utilizes a gRNA designed to complement a specific DNA sequence in the KRAS gene. The Cas9 enzyme, guided by the gRNA, introduces a double-strand break in the DNA, prompting the cell's repair machinery to introduce errors that disrupt KRAS gene function [115]. The gRNA guides the Cas9 enzyme to the target site, where it introduces a double-strand break in the DNA. The cell's repair machinery then repairs the break, often introducing errors that disrupt the function of the KRAS gene [116]. undesirable site effects refer to unintended modifications of DNA at sites other than the intended target [117]. Although CRISPR-Cas9 has been significantly improved to reduce non-specific site effects, there is still a possibility of off-target edits. Careful gRNA design, utilizing advanced algorithms, and validation of potential non-specific sites through sequencing can minimize these effects [118]. Efficient delivery of CRISPR-Cas9 components to target cells remains a challenge [119]. Methods such as viral vectors, lipid nanoparticles, and electroporation have been explored. Each approach has advantages and limitations in terms of efficiency, toxicity, and specificity [120]. The activation of the P21 protein, a well-known tumor suppressor, promotes cell cycle arrest by inhibiting cyclin-dependent kinases that regulate cell division. By halting the cell cycle, the growth of cancer cells is inhibited [121]. Activating the P21 protein through editing the KRAS gene can sensitize cancer cells to chemotherapy [122]. The increased expression of P21 leads to cell cycle arrest, which allows the chemotherapy drugs to target and kill the cancer cells more effectively [123]. Activating the P21 protein through KRAS gene editing sensitizes cancer cells to chemotherapy. Cell cycle arrest caused by P21 activation allows chemotherapy drugs to more effectively target and eliminate cancer cells [115].

The EGFR (Epidermal Growth Factor Receptor) gene plays a crucial role in cell growth, proliferation, and differentiation [124]. Mutations in the EGFR gene are associated with various cancers, particularly in NSCLC. CRISPR-Cas9 is a gene-editing technology that utilizes a guide RNA to target specific DNA sequences and the Cas9 enzyme to create double-strand breaks at the targeted location. These breaks can then be repaired, either through NHEJ or HDR, resulting in gene mutations or precise edits, respectively [125]. In the context of EGFR, CRISPR-Cas9 can be programmed to target and modify the mutated sequences responsible for cancer growth, potentially inhibiting tumor progression and improving patient outcomes [126]. Editing the EGFR gene using CRISPR-Cas9 can have both positive and negative consequences [124]. On the positive side, it can help correct mutations or deletions in the gene that are associated with certain diseases, such as lung cancer [127]. However, it is crucial to consider potential non-targeted site effects, as unintended changes in other parts of the genome could lead to unexpected consequences or disruptions in gene function [128]. The efficiency of CRISPR-Cas9 in editing the EGFR gene can vary depending on various factors, including the specific gRNA design, delivery method, and cell type [125]. Studies have shown that CRISPR-Cas9 can achieve high editing efficiency, but it is important to optimize the experimental conditions to maximize the desired outcomes [124]. CRISPR-Cas9 has high specificity, thanks to the precise binding of the guide RNA to the target DNA sequence [127]. However, there remains a concern of non-specific site effects, where Cas9 may unintentionally edit other genomic regions with partial similarity to the target site [124]. Extensive research and optimization of guide RNA design have significantly reduced undesirable site effects [127]. State-of-the-art Cas9 variants, such as high-fidelity Cas9 and base editors, have further improved specificity, minimizing the risk of unintended genetic modifications [124]. Challenges associated with CRISPR-Cas9 editing of the EGFR gene include undesirable site effects, delivery efficiency, and potential long-term effects. Ethical considerations include the need for informed consent, ensuring equitable access to the technology, and responsible use to avoid unintended consequences or the creation of "designer babies." Rigorous evaluation, regulation, and ethical guidelines are essential to navigate these challenges and ensure the responsible application of CRISPR-Cas9 in editing the EGFR gene or any other gene [128].

CRISPR-Cas9 editing of the VEGF (Vascular Endothelial Growth Factor) gene can play a crucial role in various cancers. VEGF is a protein that promotes the growth of new blood vessels, a process known as angiogenesis, which is essential for tumor development and metastasis [129]. By targeting and disrupting the VEGF gene using CRISPR-Cas9, researchers can potentially hinder the production of VEGF and, consequently, inhibit tumor angiogenesis. This could lead to reduced tumor growth and increased sensitivity to other cancer treatments [130]. While CRISPR-Cas9 is highly specific, there is a possibility of off-target effects where unintended gene edits occur. In the case of VEGF gene editing, researchers must carefully assess potential off-target sites to ensure that no critical genes are unintentionally modified [131]. To minimize undesirable site effects, rigorous bioinformatics analyses and advanced screening methods are employed to select guide RNAs with the least likelihood of non-specific site activity. CRISPR-Cas9-based VEGF gene editing, on its own, may not be sufficient for complete cancer treatment [129]. While it can impede tumor angiogenesis, a comprehensive cancer treatment strategy usually involves combining CRISPR-Cas9 with other therapies like chemotherapy, radiation, or immunotherapy [130]. Combining treatments can lead to a synergistic effect, targeting cancer cells through multiple pathways and increasing the overall therapeutic efficacy [129]. Delivering CRISPR-Cas9 components to cancer cells poses a significant challenge [131]. The large size of the Cas9 protein and guide RNA complex may limit delivery methods [131]. Various approaches are being explored, including viral vectors, nanoparticles, and liposomes, to ensure efficient and safe delivery to target cancer cells while avoiding harm to healthy tissues [129]. Ensuring long-term and stable VEGF gene suppression is essential for sustained therapeutic effects [131]. Researchers are investigating methods to improve CRISPR-Cas9 delivery and stability within cancer cells [129]. Strategies like utilizing modified Cas9 variants or integrating the CRISPR components into the genome of the target cells could potentially enhance the durability of VEGF gene editing and its anticancer effects [131].

Editing the BCL-2 gene using CRISPR-Cas9 in cancer treatment holds significant potential due to the role of the BCL-2 protein in promoting cancer cell survival [132]. By targeting and modifying the BCL-2 gene, researchers aim to disrupt the overexpression or dysregulation of this protein, which can lead to apoptosis resistance and tumor growth. CRISPR-Cas9 offers a precise and efficient method to edit the BCL-2 gene and potentially restore normal cell death mechanisms [133]. CRISPR-Cas9 utilizes guide RNAs designed to match specific sequences within the BCL-2 gene [130]. When the guide RNA finds a complementary match, it guides the Cas9 enzyme to that location, initiating a double-stranded DNA break at the target site [134]. Non-targeted site effects refer to unintended changes in DNA at sites similar to the target sequence [132]. To minimize undesirable site effects, researchers employ bioinformatic tools to carefully design guide RNAs with high specificity [134]. Additionally, thorough validation experiments are conducted to identify and mitigate any potential off-target sites [133]. BCL-2 is an anti-apoptotic gene that helps cancer cells evade cell death mechanisms [134]. By editing the BCL-2 gene, CRISPR-Cas9 can disrupt its function, promoting apoptosis in cancer cells and potentially hindering tumor growth [133]. Delivering CRISPR-Cas9 components to specific cancer cells in a patient's body poses significant challenges [132]. Researchers are exploring various delivery methods, including viral vectors and nanoparticles, to ensure efficient and targeted delivery while minimizing potential side effects [132]. Preclinical studies on animal models and in vitro experiments have shown promising results in targeting the BCL-2 gene with CRISPR-Cas9. However, clinical trials are essential to assess the safety and effectiveness of this approach in human patients [134]. Understanding the potential long-term consequences of BCL-2 gene editing is crucial. Researchers need to investigate whether edited cells retain their normal functionality and whether any unintended effects on other cellular processes occur [133]. The use of CRISPR-Cas9 in cancer treatment raises ethical questions about genetic manipulation, informed consent, and equitable access to advanced therapies [132]. Researchers and policymakers must address these concerns to ensure responsible and equitable application of this technology [134].

CRISPR-Cas9 editing of the PTEN gene can have significant effects on cancer progression [135]. PTEN is a tumor suppressor gene that regulates cell growth and division [136]. When PTEN is mutated or deleted, it leads to uncontrolled cell growth, a hallmark of cancer [137]. By using CRISPR-Cas9 to precisely target and edit the PTEN gene, researchers can potentially restore its function as a tumor suppressor, thereby inhibiting cancer cell growth and reducing tumor development [138]. The potential of CRISPR-Cas9-mediated PTEN gene editing as a treatment option varies among different cancer types [136]. Some cancers exhibit PTEN mutations as a dominant driver of tumorigenesis, making them more amenable to this approach [135]. However, the efficacy of this strategy may depend on the cancer's genetic context, as some tumors may possess alternative mechanisms to bypass PTEN function [138]. Extensive preclinical studies and clinical trials are required to determine its applicability and effectiveness across diverse cancer types [135]. Safety concerns in CRISPR-Cas9 gene editing for PTEN in cancer therapy involve potential undesirable site effects, where unintended genetic changes could occur in non-cancerous cells, leading to adverse consequences [136]. Additionally, the risk of introducing new mutations or altering other essential genes must be carefully evaluated to avoid unwanted side effects [138]. Rigorous testing in preclinical models and careful monitoring during clinical trials are crucial to ensure the safety and feasibility of this therapeutic approach [135]. Researchers are continuously exploring various strategies to improve CRISPR-Cas9 gene editing efficiency [136]. One approach involves optimizing the delivery system to ensure precise targeting of cancer cells. Additionally, advancements in CRISPR-Cas9 technology, such as using base editors or prime editors, offer more precise modifications and reduced non-targeted site effects [138]. Moreover, combining CRISPR-Cas9 with other therapies, such as immunotherapies or targeted therapies, may enhance the overall therapeutic response, allowing for a more comprehensive and effective treatment strategy. Several challenges need to be addressed when using CRISPR-Cas9 to edit the PTEN gene in cancer cells [135]. Firstly, efficient delivery of CRISPR components to specific cancer cells is crucial to avoid non-targeted site effects [138]. Secondly, ensuring the correct and precise editing of the PTEN gene without introducing unintended mutations is vital for therapeutic success [135]. Additionally, the immune response to the CRISPR components and potential immune rejection of edited cells must be evaluated to assess their long-term viability and safety [138].

The TERT gene, which encodes the telomerase reverse transcriptase enzyme, plays a critical role in maintaining telomeres, the protective caps at the ends of chromosomes [139]. In many cancer types, the TERT gene is upregulated, leading to increased telomerase activity. This allows cancer cells to bypass the natural limitations on cell division and achieve immortality, contributing to tumor growth and progression. CRISPR-Cas9 can be employed as a gene-editing tool to target and modify the TERT gene in cancer cells [140]. The CRISPR-Cas9 system consists of a guide RNA that directs the Cas9 nuclease to the desired genomic location [141]. By designing a guide RNA specific to the TERT gene sequence, researchers can guide Cas9 to the TERT gene and induce a DSB at the targeted site [142]. The cell's DNA repair machinery then repairs the break, often through the error-prone NHEJ pathway, which introduces small insertions or deletions (indels) that disrupt the TERT gene's function [139]. Alternatively, researchers can use CRISPR-Cas9 in combination with a repair template to introduce specific modifications to the TERT gene sequence, such as gene knockouts or point mutations [141]. Editing the TERT gene using CRISPR-Cas9 can lead to several outcomes. One possibility is the disruption of TERT gene function, resulting in decreased telomerase activity in cancer cells. This can lead to telomere shortening and cellular senescence or apoptosis, inhibiting the unlimited replicative potential of cancer cells [139]. Another potential outcome is the modification of TERT gene expression, such as reducing its expression level, which can hinder tumor growth [142]. Additionally, CRISPR-Cas9-mediated TERT gene editing may sensitize cancer cells to other therapies, as telomerase inhibition can enhance the effectiveness of conventional treatments like chemotherapy or radiation therapy [139].

NF-kB is a protein involved in regulating inflammation and is often overly active in various cancer types, including pancreatic cancer [143]. Researchers discovered that modifying the NF-kB gene resulted in hindering cancer cell growth and rendering the cancer cells more receptive to chemotherapy [144]. The study specifically revealed that editing the NF-kB gene suppressed the NF-kB protein, which is responsible for promoting inflammation and stimulating cell growth [145]. Consequently, this inhibition of cell growth heightened the cancer cells' sensitivity to chemotherapy since cells with subdued NF-kB protein display increased responsiveness to chemotherapy treatments [146]. Editing the NF-kB gene using CRISPR-Cas9 can have significant implications for cancer progression [143]. NF-kB is a transcription factor that plays a crucial role in regulating various cellular processes, including inflammation, cell survival, and proliferation [145]. By editing the NF-kB gene, CRISPR-Cas9 can potentially disrupt its activity, leading to the inhibition of cancer-promoting signaling pathways and the suppression of tumor growth [146]. Editing the NF-kB gene using CRISPR-Cas9 has the potential to enhance the sensitivity of cancer cells to conventional therapies [143]. NF-kB activation is often associated with resistance to chemotherapy and radiation. By disrupting NF-kB signaling through CRISPR-Cas9 editing, cancer cells may become more vulnerable to standard cancer treatments, improving overall treatment outcomes [145]. While CRISPR-Cas9 editing of the NF-kB gene shows promise, it is important to evaluate potential side effects or unintended consequences [143]. undesirable site effects, where CRISPR-Cas9 edits unintended genomic sites, could lead to genetic instability or interfere with normal cellular functions [145]. Additionally, the long-term effects of NF-kB gene disruption on overall immune response and inflammatory processes need to be thoroughly assessed [144]. Optimizing the therapeutic potential of CRISPR-Cas9 editing of the NF-kB gene requires further research and development. Understanding the specific molecular characteristics of different cancer types and their NF-kB signaling pathways is essential for designing precise CRISPR-Cas9 strategies [145]. Additionally, advancements in delivery systems, such as viral vectors or nanoparticle-based carriers, can enhance the efficiency and specificity of NF-kB gene editing in cancer cells [143].

The CDK4 gene encodes Cyclin-Dependent Kinase 4, a crucial protein involved in cell cycle regulation [147]. CDK4 forms complexes with cyclin D, leading to cell cycle progression from G1 to S phase [148]. In various cancers, the overexpression or amplification of CDK4 has been observed, promoting uncontrolled cell proliferation and tumorigenesis [149]. CRISPR-Cas9 uses guide RNA molecules that complementarily bind to the target DNA sequence within the CDK4 gene [148]. The Cas9 protein, acting as a molecular scissors, then cleaves the DNA at the precise location indicated by the guide RNA. This induces double-strand breaks in the CDK4 gene, triggering the cell's DNA repair machinery, which may lead to gene knockout or targeted mutations [149]. Preclinical studies using CRISPR-Cas9 have shown promising results in inhibiting CDK4 expression and reducing tumor growth in various cancer models, such as melanoma, breast cancer, and glioblastoma. These studies have provided valuable insights into the potential therapeutic efficacy of CRISPR-based CDK4 targeting [150]. As of the current knowledge cutoff, several clinical trials are likely underway or being planned to evaluate the safety and efficacy of CRISPR-Cas9 in editing the CDK4 gene in cancer patients. These trials will help determine the feasibility and potential benefits of CRISPR-based strategies in real-world clinical settings [151]. One challenge is the efficient delivery of CRISPR-Cas9 components to the cancer cells. Ensuring high delivery rates and minimizing non-targeted site effects is essential for successful therapy [148]. Additionally, CDK4 may have important functions in normal cells, so targeting it may cause unintended consequences in non-cancerous tissues [149].

### Clinical trials

**Fig. 7:**
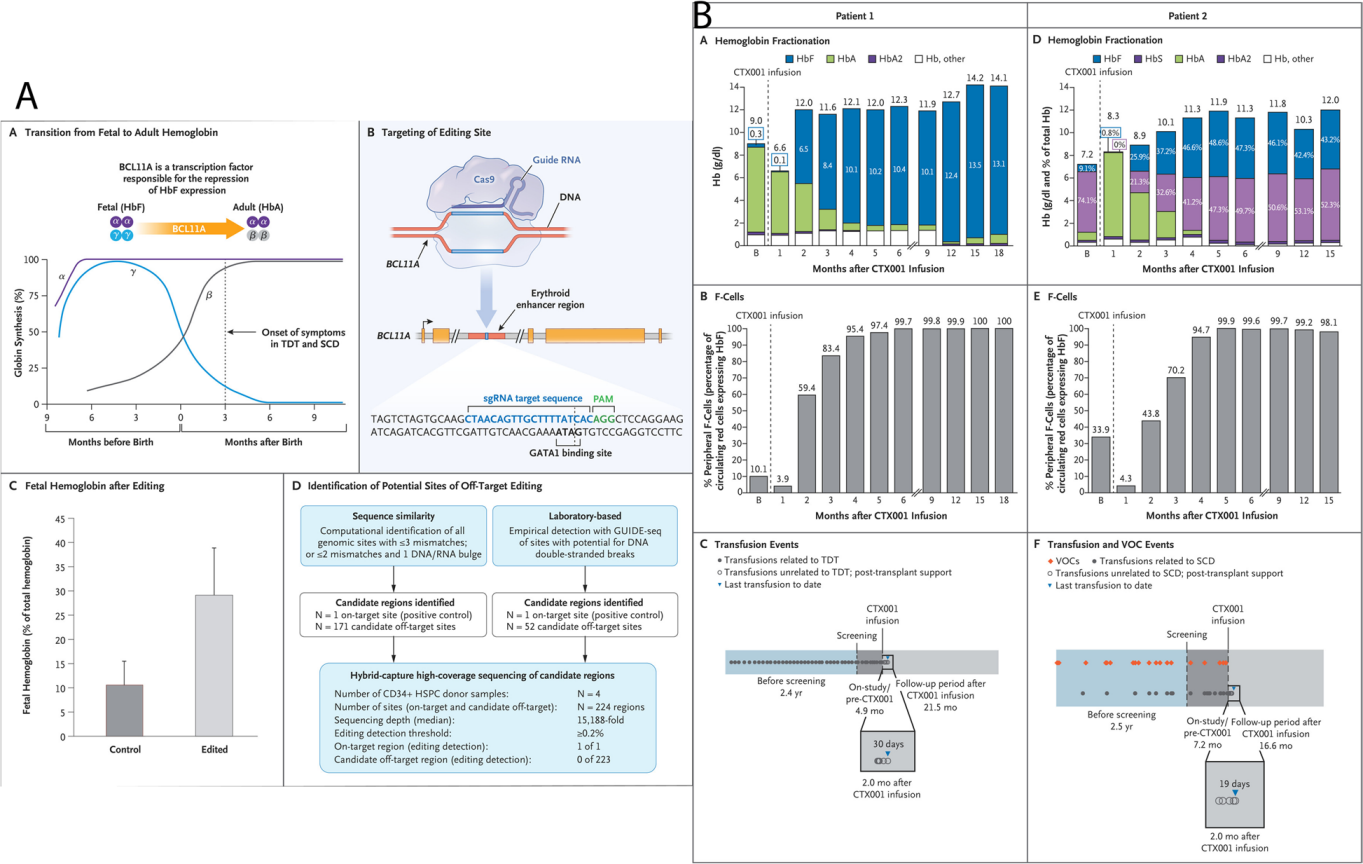
**A** The CTX001 molecular approach and preclinical studies. Panel A illustrates the shift from fetal hemoglobin (HbF) to adult hemoglobin (HbA) shortly after birth and the role of the transcription factor BCL11A in suppressing γ-globin, a component of fetal hemoglobin. When fetal hemoglobin levels decrease approximately 3 months after birth, individuals who cannot produce enough functional β-globin experience symptoms. This has implications for conditions like sickle cell disease (SCD) and transfusion-dependent β-thalassemia (TDT). Moving to Panel B, it showcases the specific editing site targeted by the single guide RNA (sgRNA) that guides CRISPR-Cas9 to the erythroid-specific enhancer region of BCL11A. The five BCL11A exons are represented as gold boxes, and GATA1 is the binding site for the GATA1 transcription factor. PAM, the protospacer adjacent motif (NGG), is a specific DNA sequence required immediately following the Cas9 target DNA sequence. Panel C displays preclinical data that reveals the percentage of fetal hemoglobin as a portion of total hemoglobin after editing and the differentiation of erythroid cells. This data was obtained from samples taken from 10 healthy donors, with error bars indicating the standard deviation. Finally, Panel D presents the results of an off-target evaluation. GUIDE-seq (genomewide unbiased identification of double-strand breaks enabled by sequencing) was independently performed on three CD34 + HSPC (hematopoietic stem and progenitor cell) healthy donor samples to nominate sites. Subsequently, hybrid capture was conducted on four CD34 + HSPC healthy donor samples to confirm these sites. The on-target allelic editing was confirmed in each experiment with an average of 57%, and no detectable off-target editing was observed at any of the sites identified by GUIDE-seq and sequence homology. Panel A was adapted with permission from Canver and Orkin. **B** The data related to hemoglobin fractionation, F-cell levels, and transfusion events in the two groups of patients under study. Panel A depicts the results of CRISPR-Cas9 treatment for transfusion-dependent β-thalassemia in Patient 1, while Panel D presents data for Patient 2, who received treatment for sickle cell disease, showcasing various hemoglobin adducts and variants. The changes in F-cell percentages over time can be observed in Panel B for Patient 1 and in Panel E for Patient 2. Baseline levels of hemoglobin and F-cells were established during the initial assessment prior to treatment. Additionally, Panel C shows the progression of transfusion events over time in Patient 1, and Panel F displays vaso-occlusive crises (VOCs) or episodes and transfusion events in Patient 2. It's worth noting that exchange transfusions performed according to the study protocol before the infusion of CTX001 during the on-study period are not included in the figures. Reprinted from [152] with permission from the New England Journal of Medicine

Clinical trials exploring the potential of CRISPR-based cancer therapy are currently in their early development stages. Nevertheless, multiple clinical trials have been launched to assess how safe and effective CRISPR-based cancer treatment is for humans. Frangoul et al.'s research is focused on the utilization of CRISPR-Cas9 gene editing to address two severe monogenic diseases [152]: Transfusion-dependent β-thalassemia (TDT) and sickle cell disease (SCD) are both severe and potentially life-threatening conditions. Researchers focused on targeting a specific transcription factor called BCL11A, which is known to inhibit the production of fetal hemoglobin and γ-globin in erythroid cells. To achieve this, they utilized the CRISPR-Cas9 system to modify the BCL11A erythroid-specific enhancer in CD34 + hematopoietic stem and progenitor cells derived from healthy donors. Remarkably, this editing approach successfully altered about 80% of the alleles at this genetic locus without any unintended undesirable site effects. After the gene editing, two patients, one with TDT and the other with SCD, underwent transplants of the edited CD34 + cells following myeloablation to remove their existing bone marrow. More than a year later, both patients exhibited significant allelic editing in their bone marrow and blood, accompanied by a substantial increase in fetal hemoglobin levels, leading to their independence from transfusions. Notably, the patient with SCD no longer experienced vaso-occlusive episodes, painful and damaging events caused by sickle-shaped red blood cells. The clinical trials for these treatments were registered on ClinicalTrials.gov with the identifiers NCT03655678 for CLIMB THAL-111 (for β-thalassemia) and NCT03745287 for CLIMB SCD-121 (for sickle cell disease). It's worth mentioning that this research received financial support from CRISPR Therapeutics and Vertex Pharmaceuticals (Fig. 7).

One common strategy observed in several trials involves knocking out the PD1 gene, which encodes for the programmed cell death protein 1. PD1 is known to be involved in inhibiting T cell activity, and by removing it, the modified T cells, known as autologous TILs and autologous EBV-specific CTLs, can become more potent at recognizing and attacking cancer cells. The Phase I clinical trials NCT03081715 [153] and NCT02793856 focus on PD1 KO Autologous TILs, while NCT03044743 [154] investigates PD1 KO Autologous EBV CTLs. Additionally, another study (NCT04417764) explores the same PD1 KO Autologous TILs strategy [155]. Another approach involves the simultaneous knockout of PD1 and the TCR gene (T cell receptor) in allogeneic mesothelin-targeting Chimeric Antigen Receptor (CAR) T cells. This strategy is being tested in the Phase I clinical trial NCT03545815 [156]. By eliminating TCR, the CAR T cells can avoid potential adverse effects like graft-versus-host disease (GVHD) while targeting mesothelin-expressing cancer cells. Furthermore, some trials focus on targeting other genes to improve CAR T cell therapy. For instance, the clinical trial NCT04037566 aims to enhance the effectiveness of CD19-targeting CAR T cells by editing the endogenous HPK1 gene [157]. Similarly, NCT04767308 utilizes endogenous CD5 knockout in allogeneic CD5-targeting CAR T cells during the early phase I trial to potentially enhance their efficacy [158]. Moreover, NCT03166878 uses the knockout of both TCR and β2m (beta-2 microglobulin) in allogeneic CD19-targeting CAR T cells [158]. The removal of TCR prevents GVHD, and the absence of β2m results in reduced expression of MHC-I, which can otherwise suppress CAR T cell activity. Other trials in Table 2 involve the insertion of CAR into T cells after knocking out specific genes. For example, NCT04502446 uses CRISPR to insert the CAR gene while simultaneously knocking out endogenous TCR and MHC-I in allogeneic CD70-targeting CAR T cells [159]. Similarly, NCT04244656 targets BCMA (B cell maturation protein) in allogeneic CAR T cells while knocking out endogenous TCR and MHC-I to boost their cancer-targeting ability [159]. Furthermore, NCT04637763 explores the knockout of PD1 and endogenous TCR in allogeneic CD19-targeting CAR T cells [159]. This approach aims to improve the persistence and activity of CAR T cells in attacking CD19-expressing cancer cells. Table 3 presents clinical trials of CRISPR-based cancer therapies targeting different cancer types.

**Table 3:** Clinical trials of CRISPR-based cancer therapy

Cancer Type	Treatment Approach	Patient Population	Description	Advantages	Disadvantages	Ref
Metastatic melanoma	TCR/CAR-T therapy targeting NY-ESO-1	Patients with NY-ESO-1 + tumors who failed prior therapy	T cells were edited to express NY-ESO-1 TCR/CAR, infused back into patients	Target specificity, long-term persistence	Possible off-target effects, limited efficacy in some patients	[160]
Non-Hodgkin's Lymphoma	CD19-targeting CAR-T therapy	Patients with refractory/relapsed NHL	T cells were edited to express CD19 CAR, infused back into patients	High response rate, durable response in some patients	Cytokine release syndrome, neurotoxicity, potential for tumor antigen escape	[161]
Bladder Cancer	PD-1 knockout via CRISPR/Cas9	Patients with high-risk non-muscle-invasive bladder cancer	CRISPR-edited autologous T cells were infused into patients	Potential for enhanced anti-tumor immune response	Off-target effects, potential for immune-related adverse events	[162]
Sarcoma	Targeted genome editing of PAX3-FOXO1 fusion gene	Patients with metastatic sarcoma expressing PAX3-FOXO1	CRISPR/Cas9 was used to target the fusion gene in tumor cells, followed by infusion of edited T cells	Specific targeting of oncogenic driver mutation	Off-target effects, limited efficacy in some patients	[163]
Solid Tumors	Adoptive transfer of TCR/CAR-T cells targeting neoantigens	Patients with advanced solid tumors	T cells were edited to express TCR/CAR targeting neoantigens unique to each patient's tumor, infused back into patients	Target specificity, potential for durable response	Heterogeneity of tumor neoantigens, potential for off-target effects	[164]
Renal Cell Carcinoma	PD-1 knockout via CRISPR/Cas9	Patients with advanced RCC who failed prior therapies	CRISPR-edited autologous T cells were infused into patients	Potential for enhanced anti-tumor immune response	Off-target effects, potential for immune-related adverse events	[165]
Multiple Myeloma	BCMA-targeting CAR-T therapy	Patients with relapsed/refractory multiple myeloma	T cells were edited to express BCMA CAR, infused back into patients	High response rate, durable response in some patients	Cytokine release syndrome, neurotoxicity	[166]
Glioblastoma	EGFRvIII-targeting CAR-T therapy	Patients with recurrent GBM expressing EGFRvIII	T cells were edited to express EGFRvIII CAR, infused back into patients	Target specificity, potential for durable response	Heterogeneity of tumor antigen expression, potential for off-target effects	[167]
Esophageal Cancer	Targeted genome editing of MUC1 gene	Patients with MUC1 + esophageal squamous cell carcinoma	CRISPR/Cas9 was used to target the MUC1 gene in tumor cells, followed by infusion of edited T cells	Specific targeting of oncogenic driver mutation	Off-target effects, limited efficacy in some patients	[168]
Prostate Cancer	Targeted genome editing of androgen receptor gene	Patients with castration-resistant prostate cancer	CRISPR/Cas9 was used to target the androgen receptor gene in tumor cells, followed by infusion of edited T cells	Specific targeting of oncogenic driver mutation	Off-target effects, limited efficacy in some patients	[169]
Acute Myeloid Leukemia	CD33-targeting CAR-T therapy	Patients with relapsed/refractory AML	T cells were edited to express CD33 CAR, infused back into patients	Target specificity, potential for durable response	Cytokine release syndrome, neurotoxicity	[170]
Head and Neck Cancer	Targeted genome editing of HPV16 E6 gene	Patients with HPV16 + recurrent or metastatic head and neck cancer	CRISPR/Cas9 was used to target the HPV16 E6 gene in tumor cells, followed by infusion of edited T cells	Specific targeting of oncogenic driver mutation	Off-target effects, limited efficacy in some patients	[171]
Lung Cancer	Targeted genome editing of KRAS gene	Patients with advanced KRAS-mutant lung cancer	CRISPR/Cas9 was used to target the KRAS gene in tumor cells, followed by infusion of edited T cells	Specific targeting of oncogenic driver mutation	Off-target effects, limited efficacy in some patients	[172]
Neuroblastoma	Targeted genome editing of ALK gene	Patients with ALK-mutant neuroblastoma	CRISPR/Cas9 was used to target the ALK gene in tumor cells, followed by infusion of edited T cells	Specific targeting of oncogenic driver mutation	Off-target effects, limited efficacy in some patients	[173]
Acute Lymphoblastic Leukemia	CD19-targeting CAR-T therapy	Pediatric patients with relapsed/refractory ALL	T cells were edited to express CD19 CAR, infused back into patients	High response rate, durable response in some patients	Cytokine release syndrome, neurotoxicity	[174]
Solid Tumors	Targeted genome editing of CCR4 gene	Patients with advanced solid tumors	CRISPR/Cas9 was used to target the CCR4 gene in tumor cells, followed by infusion of edited T cells	Specific targeting of oncogenic driver mutation	Off-target effects, limited efficacy in some patients	[175]
Melanoma	Targeted genome editing of NRAS gene	Patients with advanced NRAS-mutant melanoma	CRISPR/Cas9 was used to target the NRAS gene in tumor cells, followed by infusion of edited T cells	Specific targeting of oncogenic driver mutation	Off-target effects, limited efficacy in some patients	[176]
Cholangiocarcinoma	Targeted genome editing of IDH1 gene	Patients with advanced IDH1-mutant cholangiocarcinoma	CRISPR/Cas9 was used to target the IDH1 gene in tumor cells, followed by infusion of edited T cells	Specific targeting of oncogenic driver mutation	Off-target effects, limited efficacy in some patients	[177]
Solid Tumors	Targeted genome editing of TP53 gene	Patients with advanced solid tumors	CRISPR/Cas9 was used to target the TP53 gene in tumor cells, followed by infusion of edited T cells	Specific targeting of oncogenic driver mutation	Off-target effects, limited efficacy in some patients	[178]
Myeloma	CD19-targeting CAR-T therapy	Patients with relapsed/refractory myeloma	T cells were edited to express CD19 CAR, infused back into patients	High response rate, durable response in some patients	Cytokine release syndrome, neurotoxicity	[179]
Solid Tumors	Targeted genome editing of PD-1 gene	Patients with advanced solid tumors	CRISPR/Cas9 was used to target the PD-1 gene in tumor cells, followed by infusion of edited T cells	Enhances anti-tumor immunity by disrupting immune checkpoint pathway	Off-target effects, limited efficacy in some patients	[180]
Solid Tumors	Targeted genome editing of MUC1 gene	Patients with advanced MUC1 + solid tumors	CRISPR/Cas9 was used to target the MUC1 gene in tumor cells, followed by infusion of edited T cells	Specific targeting of oncogenic driver mutation	Off-target effects, limited efficacy in some patients	[181]
Multiple Solid Tumors	Targeted genome editing of EGFR gene	Patients with advanced EGFR-mutant solid tumors	CRISPR/Cas9 was used to target the EGFR gene in tumor cells, followed by infusion of edited T cells	Specific targeting of oncogenic driver mutation	Off-target effects, limited efficacy in some patients	[165]
Leukemia/Lymphoma	Targeted genome editing of CD22 gene	Patients with relapsed/refractory CD22 + leukemia/lymphoma	CRISPR/Cas9 was used to target the CD22 gene in tumor cells, followed by infusion of edited T cells	Specific targeting of oncogenic driver mutation	Off-target effects, limited efficacy in some patients	[182]
Solid Tumors	Targeted genome editing of PTEN gene	Patients with advanced PTEN-deficient solid tumors	CRISPR/Cas9 was used to target the PTEN gene in tumor cells, followed by infusion of edited T cells	Specific targeting of oncogenic driver mutation	Off-target effects, limited efficacy in some patients	[183]
Multiple Solid Tumors	Targeted genome editing of PDCD1 gene	Patients with advanced solid tumors	CRISPR/Cas9 was used to target the PDCD1 gene in tumor cells, followed by infusion of edited T cells	Enhances anti-tumor immunity by disrupting immune checkpoint pathway	Off-target effects, limited efficacy in some patients	[184]
Leukemia/Lymphoma	Targeted genome editing of TCR gene	Patients with relapsed/refractory leukemia/lymphoma	CRISPR/Cas9 was used to target the TCR gene in T cells, followed by infusion of edited T cells	Specific targeting of TCR gene for enhanced T-cell activity	Off-target effects, limited efficacy in some patients	[185]
Leukemia/Lymphoma	CD19-targeting CAR-T therapy	Patients with relapsed/refractory leukemia/lymphoma	T cells were edited to express CD19 CAR, infused back into patients	High response rate, durable response in some patients	Cytokine release syndrome, neurotoxicity	[186]
Solid Tumors	Targeted genome editing of HIF-1α gene	Patients with advanced HIF-1α-overexpressing solid tumors	CRISPR/Cas9 was used to target the HIF-1α gene in tumor cells, followed by infusion of edited T cells	Specific targeting of oncogenic driver mutation	Off-target effects, limited efficacy in some patients	[187]
Non-small cell lung cancer	CRISPR/Cas9-mediated PD-1 knockout	Patients with advanced PD-L1 + non-small cell lung cancer	CRISPR/Cas9 was used to knock out the PD-1 gene in T cells, followed by infusion of edited T cells	Disrupts immune checkpoint pathway	Off-target effects, limited efficacy in some patients	[188]
Multiple Solid Tumors	CRISPR/Cas9-mediated knockout of TGF-β receptor II	Patients with advanced TGF-β-overexpressing solid tumors	CRISPR/Cas9 was used to knock out the TGF-β receptor II gene in tumor cells, followed by infusion of edited T cells	Specific targeting of oncogenic driver mutation	Off-target effects, limited efficacy in some patients	[189]
Solid Tumors	CRISPR/Cas9-mediated knockout of DNMT1	Patients with advanced solid tumors	CRISPR/Cas9 was used to knock out the DNMT1 gene in tumor cells, followed by infusion of edited T cells	Specific targeting of epigenetic regulator	Off-target effects, limited efficacy in some patients	[190]
Solid Tumors	CRISPR/Cas9-mediated knockout of LAP	Patients with advanced LAP-overexpressing solid tumors	CRISPR/Cas9 was used to knock out the LAP gene in tumor cells, followed by infusion of edited T cells	Specific targeting of immunosuppressive mechanism	Off-target effects, limited efficacy in some patients	[191]
Solid Tumors	CRISPR/Cas9-mediated knockout of AXL	Patients with advanced AXL-overexpressing solid tumors	CRISPR/Cas9 was used to knock out the AXL gene in tumor cells, followed by infusion of edited T cells	Specific targeting of oncogenic driver mutation	Off-target effects, limited efficacy in some patients	[192]
Solid Tumors	CRISPR/Cas9-mediated knockout of HLA class I	Patients with advanced HLA class I-deficient solid tumors	CRISPR/Cas9 was used to knock out the HLA class I genes in tumor cells, followed by infusion of edited T cells	Specific targeting of immune evasion mechanism	Off-target effects, limited efficacy in some patients	[193]
Leukemia/Lymphoma	CRISPR/Cas9-mediated knockout of TCR and B2M	Patients with relapsed/refractory T-cell malignancies	CRISPR/Cas9 was used to knock out the TCR and B2M genes in T cells, followed by infusion of edited T cells	Disruption of T-cell receptor and MHC class I expression to prevent graft-versus-host disease and enhance anti-tumor activity	Off-target effects, limited efficacy in some patients	[194]
Solid Tumors	CRISPR/Cas9-mediated knockout of β-catenin	Patients with advanced β-catenin-overexpressing solid tumors	CRISPR/Cas9 was used to knock out the β-catenin gene in tumor cells, followed by infusion of edited T cells	Specific targeting of oncogenic driver mutation	Off-target effects, limited efficacy in some patients	[195]
Leukemia/Lymphoma	CRISPR/Cas9-mediated knockout of CD7	Patients with relapsed/refractory CD7 + leukemia/lymphoma	CRISPR/Cas9 was used to knock out the CD7 gene in T cells, followed by infusion of edited T cells	Specific targeting of B-cell antigen	Off-target effects, limited efficacy in some patients	[196]
Solid Tumors	CRISPR/Cas9-mediated knockout of PSCA	Patients with advanced PSCA-expressing solid tumors	CRISPR/Cas9 was used to knock out the PSCA gene in tumor cells, followed by infusion of edited T cells	Specific targeting of tumor-associated antigen	Off-target effects, limited efficacy in some patients	[190]
Solid Tumors	CRISPR/Cas9-mediated knockout of APOBEC3B	Patients with advanced APOBEC3B-overexpressing solid tumors	CRISPR/Cas9 was used to knock out the APOBEC3B gene in tumor cells, followed by infusion of edited T cells	Specific targeting of mutagenic enzyme	Off-target effects, limited efficacy in some patients	[197]
Solid Tumors	CRISPR/Cas9-mediated knockout of IL2RG	Patients with advanced IL2RG-deficient solid tumors	CRISPR/Cas9 was used to knock out the IL2RG gene in T cells, followed by infusion of edited T cells	Specific targeting of immunodeficiency gene	Off-target effects, limited efficacy in some patients	[198]
Solid Tumors	CRISPR/Cas9-mediated knockout of ARID1A	Patients with advanced ARID1A-mutant solid tumors	CRISPR/Cas9 was used to knock out the ARID1A gene in tumor cells, followed by infusion of edited T cells	Specific targeting of oncogenic driver mutation	Off-target effects, limited efficacy in some patients	[199]
Solid Tumors	CRISPR/Cas9-mediated knockout of TRAC	Patients with advanced TRAC-deficient solid tumors	CRISPR/Cas9 was used to knock out the TRAC gene in T cells, followed by infusion of edited T cells	Specific targeting of immunodeficiency gene	Off-target effects, limited efficacy in some patients	[200]
Solid Tumors	CRISPR/Cas9-mediated knockout of LAPTM4B	Patients with advanced LAPTM4B-overexpressing solid tumors	CRISPR/Cas9 was used to knock out the LAPTM4B gene in tumor cells, followed by infusion of edited T cells	Specific targeting of oncogene	Off-target effects, limited efficacy in some patients	[201]
Solid Tumors	CRISPR/Cas9-mediated knockout of HPRT1	Patients with advanced HPRT1-overexpressing solid tumors	CRISPR/Cas9 was used to knock out the HPRT1 gene in tumor cells, followed by infusion of edited T cells	Specific targeting of oncogene	Off-target effects, limited efficacy in some patients	[202]

## Safety and delivery challenges

CRISPR-Cas9, a revolutionary gene-editing technology, holds immense promise for the treatment of various genetic disorders and diseases [203]. However, its widespread adoption faces significant safety and delivery challenges [204]. One of the primary concerns with CRISPR-Cas9 is its potential to introduce unintended genetic changes, known as undesirable site effects, which could lead to unforeseen consequences and trigger new health problems [205]. Scientists and researchers must develop more precise and reliable methods to minimize these non-targeted site effects to ensure the safety and efficacy of the treatment [206, 207]. Additionally, delivering the CRISPR-Cas9 components into specific cells and tissues poses a significant hurdle [208, 209]. Finding efficient delivery methods that can effectively target the intended cells while avoiding adverse reactions in surrounding tissues is crucial [203]. Overcoming these safety and delivery challenges is fundamental to unlocking the full potential of CRISPR-Cas9 as a transformative therapeutic approach, offering hope for patients suffering from genetic ailments. Robust research and rigorous testing will be essential to ensure that the benefits of this groundbreaking technology outweigh any potential risks [205]. Table 4 presents the safety and delivery challenges associated with CRISPR-based cancer therapy.

**Table 4:** Safety and delivery challenges of CRISPR-based cancer therapy

Challenge	Current Approaches/Strategies	Limitations	Future Directions	Ethical and Regulatory Considerations	Description	Novelty	Advantages	Disadvantages	Limitations/Challenges	Ref
Off-Target Effects	Targeted Genome Editing	- Can be difficult to achieve high specificity	- Development of improved delivery methods and monitoring techniques	- Need for clear guidelines and oversight	CRISPR can cause unintended changes to the genome outside the target region, which can lead to adverse effects	CRISPR-mediated genome editing is a highly precise and targeted approach to gene therapy	- Minimizes damage to healthy cells	- Possibility of unintended changes to the genome	Specificity needs to be improved to avoid off-target effects	[206, 207]
Delivery to Tumor Site	Use of Viral Vectors	- Lack of specificity, difficulty targeting tumor cells	- Development of targeted delivery systems	- Need to minimize risk to healthy tissues	Viral vectors are commonly used to deliver CRISPR-based therapies, but can be difficult to target to specific tumor cells	Targeted delivery of CRISPR-based therapies can minimize damage to healthy tissues	- Can be used to target specific tumor cells	- Can cause immune reactions and toxicity	Targeting specificity needs to be improved to avoid damage to healthy tissues	[208, 209]
Safety	Monitoring Off-Target Effects	- Limited sensitivity and accuracy of current monitoring techniques	- Development of more sensitive and accurate monitoring techniques	- Need to ensure patient safety and minimize potential harm	Monitoring of off-target effects is essential to ensure the safety of CRISPR-based therapies	Improved monitoring techniques can provide more accurate information about the effects of CRISPR editing on the genome	- Can help to detect unintended changes to the genome	- Current techniques have limited sensitivity and accuracy	More research is needed to improve monitoring techniques	[210, 211]
Immune Reactions	Use of Non-Immunogenic CRISPR Systems	- Limited availability of non-immunogenic CRISPR systems	- Development of non-immunogenic CRISPR systems	- Need to minimize the risk of immune reactions and toxicity	CRISPR-based therapies can trigger immune responses, which can lead to adverse effects	Non-immunogenic CRISPR systems are being developed to minimize the risk of immune reactions	Non-immunogenic CRISPR systems can reduce the risk of immune reactions and toxicity	- Non-immunogenic CRISPR systems are not widely available	More research is needed to develop non-immunogenic CRISPR systems	[212, 213]

### Delivery challenges and safety measures in CRISPR-based gene editing

The evaluation of off-target effects in gene editing is crucial to ensuring the safety and precision of genetic modifications [214]. Advancements in base editors and prime editors aim to minimize non-selective site effects, but ongoing research and ethical considerations are necessary to harness these technologies responsibly for therapeutic applications and other genetic interventions [215]. Off-target effects refer to unintended changes or alterations in the DNA that occur when using gene editing technologies like CRISPR-Cas systems. These unintended modifications can happen at sites other than the targeted location, potentially leading to unpredictable and unwanted genetic changes [214]. Base editors are a recent advancement in CRISPR technology that can perform targeted chemical modifications to specific DNA bases without creating double-stranded breaks like traditional CRISPR-Cas systems. This targeted approach reduces the risk of undesirable site effects by minimizing the potential for random DNA alterations [215]. Prime editors offer enhanced precision in gene editing compared to base editors or traditional CRISPR-Cas systems. They combine the capabilities of base editors and nickases, allowing for accurate insertion, deletion, and substitution of particular genetic bases within the genome. This increased precision further reduces the likelihood of non-targeted site effects [216]. Scientists use various techniques to assess non-selective site effects, such as whole-genome sequencing, high-throughput sequencing, and computational analysis. These methods help identify unintended genetic changes and determine the efficiency and specificity of the gene-editing technology being used [217]. Despite the advancements in base editors and prime editors, off-target effects remain a concern [217]. The challenge lies in achieving absolute precision in targeting specific genomic sites without affecting nearby regions [215]. Continuous refinement of gene-editing tools, along with rigorous evaluation and validation methods, are crucial to overcoming these challenges [216]. To ensure the safety of using base editors and prime editors in therapeutic settings, comprehensive preclinical studies are necessary. These studies involve rigorous testing of the gene-editing tools on relevant cell lines and animal models to assess potential non-targeted site effects and ensure the accuracy of genetic modifications before progressing to human trials [215].

**Fig. 8:**
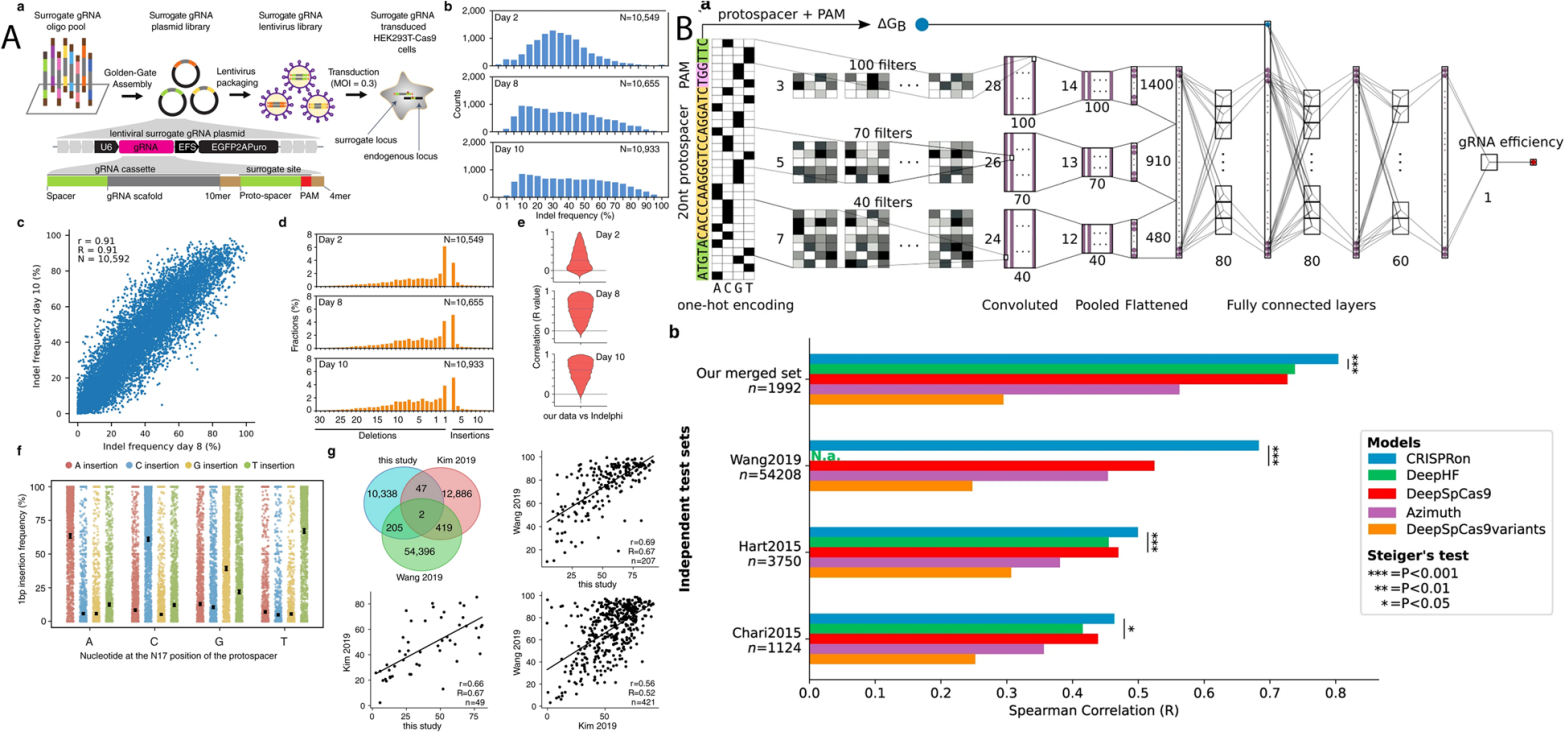
**A** The process and results of high-throughput quantification of gRNA efficiency in cells. In panel (a), a graphic illustrates the sequence of actions involved, which includes employing a lentiviral surrogate vector, synthesizing an oligo pool, performing PCR amplification, using golden-gate assembly, packing the genetic material into lentiviruses, and then introducing it. Panel (b) showcases the editing efficiency of gRNA at all surrogate locations, assessed through targeted amplicon sequencing. The results are presented for HEK293T-SpCas9 cells at 2, 8, and 10 days following the introduction. Panel (c) displays the correlation between gRNA editing efficiency on days 8 and 10 post-transduction. Panel (d) presents the patterns of indels (deletions ranging from 1–30 bp and insertions ranging from 1–10 bp) introduced by SpCas9 in HEK293T-SpCas9 cells at 2, 8, and 10 days after the transduction. Panel (e) depicts the agreement between the observed indel patterns in cells and those predicted by inDelphi, visualized as a violin plot with medians and quartiles. In panel (f), a scatter plot portrays the frequency of 1-bp insertion indels (mean ± 95% confidence interval), categorized based on the nucleotide at position N17 of the protospacer and the type of inserted nucleotide. Lastly, panel (g) exhibits the association between gRNA editing efficiencies in this study and those from other significant research, with a particular emphasis on common gRNA + PAM (23 nt) cases, presented using a Venn diagram. **B** The CRISPR on model and its ability to generalize on independent test sets. Panel a displays a visual depiction of the input DNA sequence for CRISPRon, including the prediction algorithm. The deep learning network receives inputs in the form of a one-hot encoded 30mer and the binding energy (ΔGB). It's worth noting that only the filtering (convolutional) layers and the three fully connected layers are explicitly depicted, with the thin vertical bars representing the output of one layer, serving as the input for the next layer. In panel b, a performance evaluation comparing CRISPRon to other existing models is presented, specifically focusing on independent test sets containing over 1000 gRNAs. Reprinted from [223] with permission from Springer Nature

One strategy is to use high-fidelity Cas9 variants, which have been engineered to have reduced off-target activity [215]. Another strategy is to use alternative CRISPR systems, such as Cpf1 or Cas12a, which have unique mechanisms of target recognition and have shown to have lower off-target activity compared to Cas9 [218]. Another strategy is to use a combination of gRNA and Cas9 variants with high specificity, or to use multiple gRNAs to target the same gene. This increases the specificity of the CRISPR-based gene editing and reduces the risk of non-targeted site effects [219]. Another way to minimize off-target effects is to use computational tools to predict potential undesirable site sites and to experimentally validate these predictions. This allows researchers to identify and avoid non-selective site sites before they cause unintended mutations [220]. Finally, it is important to note that off-target effects can also arise from the delivery method used to deliver the CRISPR machinery to the cells [221]. Researchers are developing a variety of methods to deliver CRISPR to the tumor site, including viral vectors, nanoparticles, and exosomes [222]. Research by Xiang et al. focuses on improving the efficiency prediction of CRISPR-Cas9 gRNAs using data integration and deep learning (Fig. 8). The primary aim is to enhance the accuracy of identifying gRNAs that will be more effective in targeting specific DNA sequences. CRISPR-Cas9 is a powerful gene-editing tool that relies on gRNAs to guide the Cas9 enzyme to the target DNA site for editing. Efficient gRNA design is crucial for successful genome editing, and this requires reliable predictions of on-target efficiency. To achieve this, the researchers gathered high-quality gRNA activity data for 10,592 gRNAs that target the SpCas9 enzyme. To further improve their predictions, the researchers integrated this new data with existing complementary data from other sources. They then employed a deep learning model called "CRISPR on," which was trained on a combined dataset of 23,902 gRNAs, including both the newly generated data and the previously available data. The results of their study showed that CRISPR on outperformed existing tools used for gRNA efficiency prediction. The improved performance was observed across four test datasets that were not part of the training data used for developing other prediction tools. This suggests that CRISPR on's predictions were more accurate and reliable than what was currently available. To make their findings accessible to the scientific community, the researchers developed an interactive webserver for gRNA design based on the CRISPR on standalone software. This webserver allows researchers to easily access and use the CRISPR on tool for designing gRNAs with higher efficiency [223]. However, these delivery methods are still in early stages of development and more research is needed to optimize their effectiveness and safety [224]. Undesirable site effects are a major concern in CRISPR-based gene editing, and they are particularly relevant in the context of cancer therapy [224]. Researchers are developing new strategies to minimize the risk of non-selective site effects, including using high-fidelity Cas9 variants, alternative CRISPR systems, computational tools, and optimized delivery methods [224, 225]. While these strategies have shown promise, much work remains to be done to ensure that CRISPR-based gene editing is safe and effective for cancer therapy [226]. Non-targeted site effects refer to unintended changes in the DNA of cells caused by the CRISPR-Cas9 system. These changes can occur in genes that were not intended to be targeted by the CRISPR-Cas9 system. These unintended changes can compromise the therapeutic effect of gene editing and potentially lead to harmful consequences [227]. Researchers used CRISPR-Cas9 to target the PIK3CA gene in human cancer cells. PIK3CA is a well-established oncogene, or a gene that promotes the development of cancer. The researchers found that the CRISPR-Cas9 system caused unintended mutations in several non-targeted site genes, including the AKT1 gene. These undesirable site effects could have compromised the therapeutic effect of targeting PIK3CA, as these mutations could activate AKT1 and thus promote cancer growth [228]. Similarly, other researchers used CRISPR-Cas9 to target the KRAS gene in human cancer cells. KRAS is also a well-established oncogene. Investigators found that the CRISPR-Cas9 system caused unintended mutations in several off-target genes, including the NF1 gene. These non-selective site effects could have compromised the therapeutic effect of targeting KRAS, as these mutations could inactivate NF1 and thus promote cancer growth [229]. Researchers used CRISPR-Cas9 to target the MYC gene in human cancer cells. MYC is a well-established oncogene. They found that the CRISPR-Cas9 system caused unintended mutations in several off-target genes, including the BCL2L11 gene. These undesirable site effects could have compromised the therapeutic effect of targeting MYC, as these mutations could activate BCL2L11 and thus promote cancer growth [230]. Other researchers used CRISPR-Cas9 to target the TERT gene in human cancer cells. TERT is a gene that promotes the growth of cancer. They found that the CRISPR-Cas9 system caused unintended mutations in several off-target genes, including the NFE2L2 gene. These non-targeted site effects could have compromised the therapeutic effect of targeting TERT, as these mutations could inactivate NFE2L2 and thus promote cancer growth [139]. The purpose of using CRISPR-Cas9 was to investigate the potential therapeutic effect of targeting the BRCA1/TP53/RAS gene, which is commonly mutated in breast and ovarian cancer/cancer [231]. The unintended consequences of using CRISPR-Cas9 on the BRCA1/TP53/RAS gene were unintended mutations in several off-target genes, including RAD51D/MDM2/MAPK, respectively [232]. The unintended mutations in RAD51D/MDM2/MAPK genes could have compromised the therapeutic effect of targeting BRCA1/TP53/RAS as they could activate RAD51D/ inactivate MDM2/activate MAPK, leading to the promotion of cancer growth [233]. The frequent findings of unintended mutations in undesirable site genes in these studies highlight the significant risk of non-selective site effects associated with CRISPR-Cas9 gene editing [234]. The research should analyze and compare the off-target effects observed when targeting different genes, which could provide insights into the gene-specific effects of CRISPR-Cas9 [235, 236]. Understanding the off-target effects and their potential impact on cancer growth is crucial in assessing the safety and efficacy of CRISPR-Cas9 as a therapeutic approach for cancer treatment [232, 237].

### Safety

Safety is a critical concern in the development of CRISPR-based cancer therapy [238]. CRISPR-based gene editing has the potential to cause unintended mutations in the genome, which can lead to serious side effects. This is particularly concerning in cancer therapy, where the goal is to target specific genetic mutations that drive tumor growth [239]. One of the main safety concerns with CRISPR-based cancer therapy is the potential for non-targeted site effects. This occurs when the CRISPR machinery targets unintended genes, leading to unintended mutations [240]. Researchers are working to develop more precise CRISPR delivery methods and to improve the specificity of the guide RNAs used to target specific genes [241]. Newer versions of CRISPR- Cas12a and Cas13 for example, have a higher specificity than the original Cas9, which helps in reducing undesirable site effects [242]. Another safety concern is the possibility of creating new cancer-causing mutations [241]. CRISPR-based cancer therapy relies on the ability to precisely target specific genetic mutations that drive tumor growth [238]. However, if the CRISPR machinery inadvertently targets other genes, it could create new cancer-causing mutations [243]. To mitigate this risk, researchers are developing new strategies to minimize the risk of non-specific site effects and to better understand the long-term effects of CRISPR-based cancer therapy [238]. A third safety concern is the risk of the edited cells becoming cancerous [240]. CRISPR-based cancer therapy relies on the ability to edit specific genetic mutations that drive tumor growth. However, if the edited cells acquire additional mutations, they could become cancerous [238]. Researchers are working to understand the long-term effects of CRISPR-based cancer therapy and to develop strategies to minimize the risk of the edited cells becoming cancerous [241]. In addition to these concerns, CRISPR-based cancer therapy raises a number of other safety concerns, including the potential for immune reactions to the viral vectors used to deliver CRISPR, and the risk of creating new cancer-causing mutations. To mitigate these concerns, researchers are developing safer delivery methods and developing new strategies to minimize the risk of non-targeted site effects [240]. Preclinical and clinical studies are being conducted to evaluate the safety and efficacy of CRISPR-based cancer therapy [238]. Additionally, researchers are working to find cost-effective and efficient methods for producing large numbers of CRISPR-edited cells, and to overcome scalability issues [240]. CRISPR-based gene editing has the potential to revolutionize cancer therapy, but significant safety challenges remain to be addressed before this approach can be safely and effectively used in the clinic [241]. Ongoing research is essential to better understand the long-term effects of CRISPR-based cancer therapy, to develop safer delivery methods, and to minimize the risk of non-targeted site effects and other safety concerns [240]. Another aspect of safety in CRISPR-based cancer therapy is the delivery method used to deliver the CRISPR machinery to the tumor cells. One of the most commonly used methods is the use of viral vectors, such as adenoviruses or lentiviruses [241]. However, these vectors have the potential to cause immune reactions and other adverse effects [238]. Researchers are working on developing non-viral delivery methods, such as nanoparticles and exosomes, as an alternative to viral vectors. These methods have the potential to be safer and more effective, but they are still in early stages of development and more research is needed to optimize their effectiveness and safety [241, 244]. Additionally, the manufacturing and scalability of CRISPR-based cancer therapy is another important safety concern. Producing large quantities of CRISPR-modified cells for clinical use is challenging and costly [241]. Researchers are working to find cost-effective and efficient methods for producing large numbers of CRISPR-edited cells, and to overcome scalability issues. This includes researching alternative methods of producing the CRISPR machinery and exploring ways to improve the efficiency of the CRISPR editing process [241]. Safety is a critical concern in the development of CRISPR-based cancer therapy [240]. Researchers are working to address these concerns by developing safer delivery methods, developing new strategies to minimize the risk of non-targeted site effects and other safety concerns, and finding cost-effective and efficient methods for producing large numbers of CRISPR-edited cells [238]. Ongoing research is essential to better understand the long-term effects of CRISPR-based cancer therapy, and to ensure that this promising new approach can be safely and effectively used in the clinic. It's important to note that while CRISPR-based cancer therapy is a promising new approach, it is still in the early stages of development. Many of the safety concerns and challenges discussed above are still being studied and evaluated in preclinical and clinical trials. Therefore, it is important to continue monitoring the progress of research in this field and to evaluate the safety and efficacy of CRISPR-based cancer therapy as more data becomes available [240]. It is also worth noting that the regulatory landscape for CRISPR-based cancer therapy is still evolving. Different countries and regions have different regulations and guidelines regarding the use of CRISPR-based therapies in humans [241]. Researchers and companies developing CRISPR-based cancer therapies will need to navigate these regulations and guidelines in order to bring their therapies to market [238]. The CRISPR-based cancer therapy has the potential to revolutionize cancer treatment, but significant safety challenges and delivery issues still need to be addressed. Researchers are working to address these concerns through ongoing research and development, but it will take time to fully understand the long-term effects and safety of this new approach. It is important to monitor the progress of research in this field and to evaluate the safety and efficacy of CRISPR-based cancer therapy as more data becomes available [241].

### Delivery to the tumor site

Delivery of CRISPR-based gene editing to the tumor site is a major challenge in the development of CRISPR-based cancer therapy [245]. CRISPR-based therapy uses a viral vector, nanoparticles, or exosomes to deliver the CRISPR machinery to the tumor cells [245]. The delivery method used is crucial for the efficiency of the therapy, as well as the safety of the patient [246]. One of the most commonly used methods to deliver CRISPR to the tumor site is through the use of viral vectors [247]. Viral vectors are modified versions of viruses that can be used to introduce genes or other genetic material into cells [248]. The most commonly used viral vectors for CRISPR delivery are adeno-associated viruses (AAVs) and lentiviruses. These vectors have been shown to efficiently deliver CRISPR to a variety of cells, including cancer cells [249]. However, the use of viral vectors raises safety concerns, as the immune system may recognize the virus as foreign and mount an immune response. This can lead to inflammation and other adverse effects, and can also limit the effectiveness of the therapy [250]. Another method to deliver CRISPR to the tumor site is through the use of nanoparticles. These particles are small enough to easily penetrate the tumor tissue, and can be engineered to carry the CRISPR machinery [251]. Nanoparticles can also be designed to target specific cell types, such as cancer cells, to increase the efficiency of the therapy [252]. However, the efficacy of nanoparticles in delivering CRISPR to the tumor site is still being evaluated, and more research is needed to understand their safety and effectiveness [253]. Exosomes are also considered as a promising delivery method for CRISPR [254]. Exosomes are small vesicles that are naturally released by cells and can be engineered to carry CRISPR machinery [255]. Exosomes have the ability to cross the blood–brain barrier and deliver the CRISPR machinery to the tumor site. But more research is needed to understand the safety and efficacy of exosomes as a delivery method for CRISPR [256]. Researchers are developing CRISPR delivery methods that can target cancer cells based on surface markers, such as the expression of specific proteins [257]. Another approach is to target genetic mutations that are specific to cancer cells. This can be done by engineering the CRISPR machinery to recognize and target specific genetic sequences associated with cancer [255]. For example, researchers have developed CRISPR-based therapies that target specific mutations in genes such as KRAS, which is commonly mutated in many types of cancer [256]. A combination of these strategies to target specific cell types can also be used to deliver CRISPR to the tumor site [248]. For example, researchers are exploring the use of nanoparticles that are designed to target specific surface markers on cancer cells and also carry the CRISPR machinery [245]. Delivery of CRISPR to the tumor site is a critical step in the development of CRISPR-based cancer therapy [248]. Researchers are working to develop new and efficient methods for delivering CRISPR to the tumor site, including viral vectors, nanoparticles, and exosomes [251]. Additionally, targeting specific cell types, such as cancer cells, can increase the efficiency of the therapy. While significant challenges remain to be addressed, the potential of CRISPR-based cancer therapy to revolutionize cancer treatment is clear, and ongoing research will help to overcome these challenges [255].

### Manufacturing and scalability

CRISPR-based gene editing has the potential to revolutionize cancer therapy by precisely targeting genetic mutations that drive tumor growth [258]. However, significant challenges remain to be addressed before this approach can be safely and effectively used in the clinic [200]. One of the major challenges is the manufacturing and scalability of CRISPR-modified cells for clinical use [259]. Manufacturing CRISPR-edited cells for use in cancer therapy is a complex and costly process. The first step is to obtain the cells that will be edited, which can be obtained from the patient or from a cell line. Once the cells are obtained, they must be modified using the CRISPR machinery. This typically involves delivering the CRISPR machinery, including the guide RNAs and Cas enzymes, to the cells using a viral vector or nanoparticle [260]. However, this process is not yet fully optimized and more research is needed to find efficient and cost-effective methods for producing large numbers of CRISPR-edited cells [261]. Scalability is also a major challenge in the manufacture of CRISPR-edited cells for cancer therapy [262]. The current methods for producing CRISPR-edited cells are not yet able to produce the large numbers of cells required for clinical use [258]. For example, if the cells are produced using a viral vector, the process is limited by the number of cells that can be infected at one time [263]. Additionally, the current methods for producing CRISPR-edited cells are not yet able to produce cells with a high enough efficiency to be clinically relevant [264]. There are a number of potential solutions to these challenges. Researchers are working to develop more efficient and cost-effective methods for producing CRISPR-edited cells, such as using exosomes as a delivery method, and to improve the scalability of the process [265]. Additionally, researchers are working to improve the efficiency of the CRISPR-editing process and to minimize the risk of non-targeted site effects [200]. Another potential solution to the scalability challenge is the use of cell lines that have been genetically engineered to produce high numbers of CRISPR-edited cells [259]. For example, researchers have developed cell lines that stably express Cas enzymes, which can be used to produce large numbers of CRISPR-edited cells [260]. Additionally, researchers are exploring the use of stem cells as a source for CRISPR-edited cells [264]. Stem cells have the ability to self-renew and differentiate into a wide range of cell types, making them an attractive option for producing large numbers of CRISPR-edited cells for cancer therapy. Another area of active research is the development of automated platforms for producing CRISPR-edited cells. These platforms can automate many of the manual steps involved in the production of CRISPR-edited cells, making the process more efficient and cost-effective [200]. Additionally, these platforms can be used to optimize the conditions for producing CRISPR-edited cells, such as the amount of Cas enzymes and guide RNAs used [259]. Finally, researchers are exploring the use of in situ delivery of CRISPR machinery, which allows the cells to be edited directly in the tumor microenvironment [261]. This approach avoids the need to produce and deliver large numbers of CRISPR-edited cells, and could potentially overcome the scalability challenges [264]. However, this approach is still in the early stages of development and more research is needed to optimize its effectiveness and safety [260]. One example is the use of exosomes as a delivery method for CRISPR machinery [264]. Exosomes are small vesicles that are naturally released by cells and can be used to deliver a variety of molecules, including CRISPR machinery, to target cells [261]. Researchers have shown that exosomes can be used to deliver CRISPR machinery to cancer cells with high efficiency and minimal toxicity. This approach is being further developed as a potential solution to the scalability challenge. Another example is the use of cell lines that have been genetically engineered to produce high numbers of CRISPR-edited cells [264]. Researchers have developed cell lines that stably express Cas enzymes and can be used to produce large numbers of CRISPR-edited cells. This approach is being further developed as a potential solution to the scalability challenge. Another example is the development of automated platforms for producing CRISPR-edited cells. These platforms automate many of the manual steps involved in the production of CRISPR-edited cells, making the process more efficient and cost-effective. This approach is being further developed as a potential solution to the scalability challenge [200]. Finally, there are examples of research on in situ delivery of CRISPR machinery [260]. Researchers have developed methods for delivering CRISPR machinery directly to cancer cells in the tumor microenvironment [261]. This approach avoids the need to produce and deliver large numbers of CRISPR-edited cells, and could potentially overcome the scalability challenges [264]. This approach is still in the early stages of development and more research is needed to optimize its effectiveness and safety [259]. The manufacturing and scalability of CRISPR-modified cells for clinical use is a major challenge that needs to be overcome for CRISPR-based cancer therapy to become a viable clinical option [261]. Researchers are working to develop more efficient and cost-effective methods for producing CRISPR-edited cells, such as using exosomes as a delivery method, and improving scalability by using genetically engineered cell lines or stem cells, and by developing automated platforms and in situ delivery method [200].

## Conclusion and future directions

The investigation into CRISPR-based gene editing for cancer treatment, as elaborated in this thorough analysis, marks a significant paradigm shift in our strategies for fighting cancer. The capabilities of CRISPR in tackling the intricate characteristics of cancer via targeted genomic alterations are substantial. Approaches including deactivating genes that promote tumor growth, boosting the body's immune reaction to cancer cells, correcting genetic anomalies that lead to cancer, and attacking tumors directly with toxic agents, have all indicated promising pathways in the realm of cancer therapeutics. Early-stage research and clinical experiments have started to reveal the effectiveness and transformative potential of CRISPR in the context of cancer treatment. These investigations have not only yielded hopeful outcomes but have also clarified the trajectory ahead. Yet, there are notable obstacles to overcome. The accuracy of CRISPR, its greatest advantage, raises concerns about unintended genetic impacts, known as off-target effects. The paramount importance lies in ensuring the security and precision of CRISPR interventions, necessitating continuous research to address these concerns. The delivery of CRISPR components to tumor cells presents another significant challenge. Developing methods that are both effective and safe for delivering these components is vital for the practical use of CRISPR in treating cancer. This challenge is heightened by the variability of tumor types and the complexity inherent in human biology. Nonetheless, the prospective future of CRISPR in cancer treatment is exceptionally promising. With ongoing advancements in research surmounting existing barriers, there is a tangible possibility for the creation of more efficient, individualized, and minimally invasive treatments for cancer. Such advancements could fundamentally transform the approach to cancer care, moving from traditional chemotherapy and radiation to specific genetic treatments. The CRISPR methodology presents an innovative and potentially game-changing strategy in cancer therapy. The journey ahead is laden with hurdles, such as ensuring the safety, accuracy, and efficient delivery of treatments. Despite these challenges, the progress achieved to date is promising. Continuous investigation and development in this area are crucial to fully harness the capabilities of CRISPR-based therapies in combating cancer. Looking forward, it is vital to confront these challenges directly, concentrating on refining methods, improving delivery systems, and prioritizing patient safety, in order to fully exploit the revolutionary potential of this technology in cancer care.

**Fig. 9:**
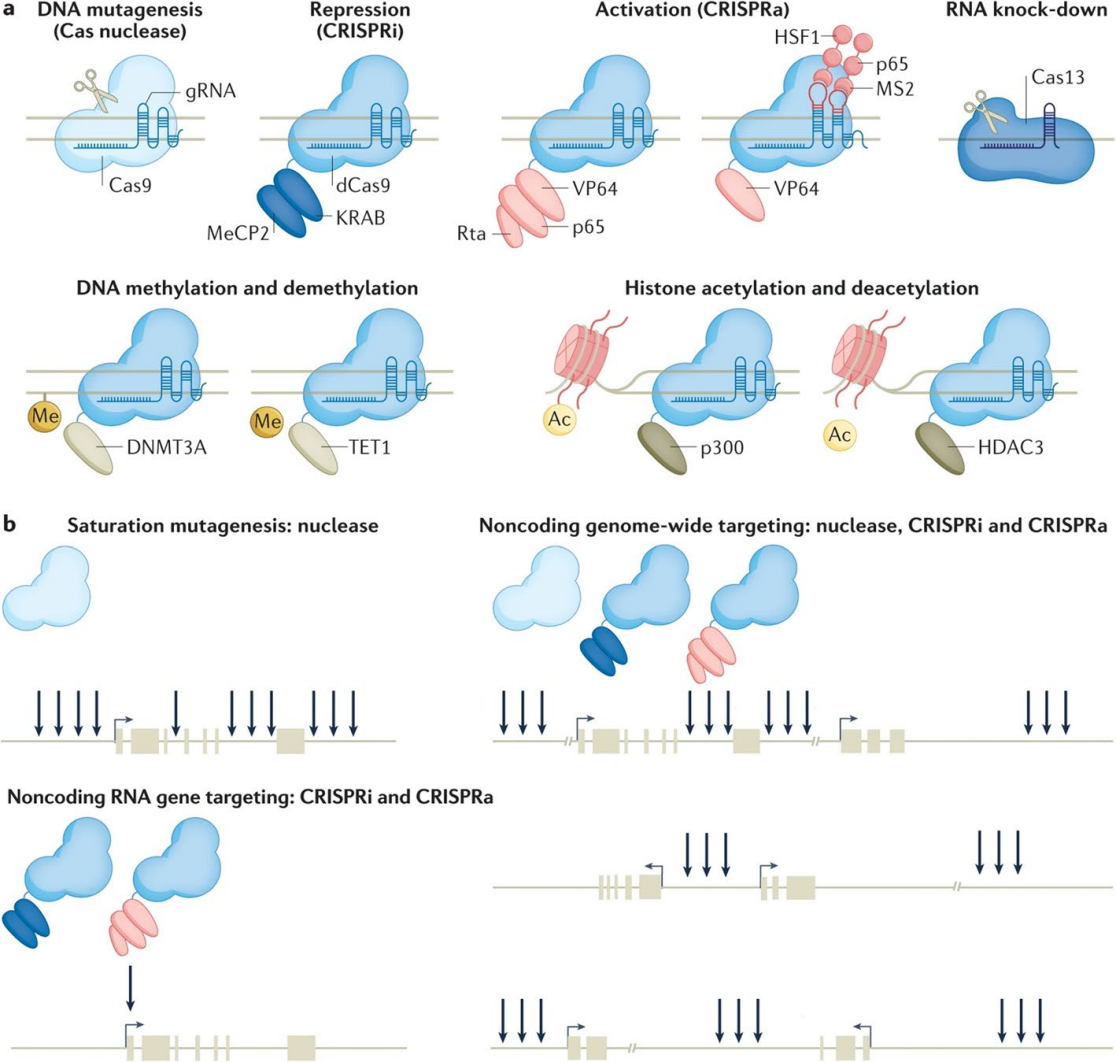
The functional domains of different CRISPR effectors and their applications in genome-scale screens. Multiple CRISPR effectors are accessible for disrupting coding and noncoding DNA and RNA segments. One commonly employed CRISPR effector is the CRISPR-associated 9 (Cas9) nuclease, which precisely cuts DNA at a specified target site guided by a guide RNA (gRNA). Noncoding regions can be suppressed with CRISPR interference (CRISPRi) by directing the catalytically inactive Cas9 (dCas9) to promoters and enhancer regions. It can be fused with repressor domains like methyl-CpG-binding protein 2 (MeCP2) and Krüppel-associated box (KRAB). Conversely, gene expression can be enhanced by directing dCas9 fusion proteins to regions around transcription start sites (TSSs). One approach is to fuse dCas9 with transcriptional activators such as VP64, p65, and Rta (VPR). Another method is fusing dCas9 with VP64 and using a modified single gRNA (sgRNA) to recruit the activator fusion complex MS2–p65–HSF1, collectively known as the synergistic activation modulator (SAM). In addition to targeting DNA, the Cas13 nuclease can be employed to cleave RNA at a specific site indicated by a gRNA. Furthermore, dCas9 can be combined with methyltransferases (e.g., DNA methyltransferase 3A or DNMT3A) to enable targeted DNA methylation or with proteins involved in DNA demethylation (e.g., tet methylcytosine dioxygenase 1 or TET1) to facilitate targeted DNA demethylation. Moreover, linking dCas9 to acetyltransferases like p300 or histone deacetylase proteins like histone deacetylase 3 (HDAC3) enables targeted histone acetylation or deacetylation, respectively. The design of gRNAs depends on the specific CRISPR effector and the intended targets of the CRISPR screen. When focusing on protein-coding genes, gRNAs can be designed to target either exons (using CRISPR nucleases) or regions near the transcription start site (TSS) of the gene (for CRISPRi or CRISPR activation (CRISPRa)). For saturation mutagenesis using nucleases, gRNAs are designed to target multiple noncoding regions around a gene of interest. In noncoding genome-wide screens using CRISPR nucleases, CRISPRi, or CRISPRa, gRNAs are tailored to specific genomic features like cis-regulatory elements. When silencing or amplifying noncoding RNAs with CRISPRi and CRISPRa, respectively, sgRNAs are directed to regions flanking the transcription start site (TSS) of a noncoding RNA gene. Reprinted from [11] with permission from Springer Nature

The future of CRISPR-based cancer therapy is promising, with vast potential for personalized and effective treatments, but it requires multidisciplinary efforts, ethical considerations, and international collaboration to ensure its successful translation into clinical practice [266]. Table 5 presents the future directions for CRISPR-based cancer therapy. Moreover, exploring non-coding regions of the genome and applying CRISPR screens to identify new therapeutic targets offer promising avenues for treatment advancements. Figure 9 illustrates the different functional aspects of various CRISPR effectors and their applications in genome-scale screens. The integration of CRISPR technology with emerging imaging and sensing technologies can enhance the monitoring and tracking of treatment outcomes [267]. Collaborative efforts between academia and industry will accelerate drug development and foster more efficient translational opportunities [266]. Furthermore, combining CRISPR-based interventions with other therapies in a synergistic approach warrants exploration, while CRISPR-based diagnostic tools will aid in early cancer detection and diagnosis [268]. Gene editing technology can be leveraged to develop personalized cancer vaccines and improve immunotherapy response. In terms of societal impact, there is a need for equitable access to CRISPR-based cancer therapy to ensure all patients benefit from these advancements, regardless of their background or location. This calls for international cooperation in establishing regulatory frameworks for gene editing technology and promoting public understanding and acceptance of these therapies through education and outreach [269]. Other research priorities involve studying the effects of CRISPR gene editing on the tumor microenvironment and immune system, addressing genetic discrimination and privacy concerns related to gene editing, and evaluating the long-term safety and efficacy of CRISPR-based cancer therapy [18]. Lastly, developing CRISPR gene editing systems for rare or difficult-to-treat cancers, harnessing nanotechnology for targeted delivery of CRISPR, and establishing databases for data sharing are critical for pushing the boundaries of CRISPR-based cancer therapy [270]. Public–private partnerships and collaboration between clinicians and researchers are instrumental in optimizing therapy design and delivery for better patient outcomes [267].

**Table 5:** Future directions for CRISPR-based cancer therapy

Research Priorities	Emerging Technologies	Translational Opportunities	Collaborative Efforts	Societal Impact	Ref
Development of more precise and efficient delivery systems	Expansion of genome editing tools beyond gene knockout and correction	Development of biomarkers to predict treatment response	Collaborative development of ethical guidelines for CRISPR-based therapy	Equitable access to CRISPR-based cancer therapy	[268]
Development of gene editing tools to target non-coding regions of the genome	Application of CRISPR screens to identify new therapeutic targets	Use of gene editing to improve immunotherapy response	Collaboration between academic institutions and industry to accelerate drug development	Impacts on healthcare economics and resource allocation	[269]
Exploration of combination therapies that include CRISPR-based interventions	Integration of CRISPR technology with emerging imaging and sensing technologies	Application of CRISPR gene editing to develop personalized cancer vaccines	Collaborative development of CRISPR-based diagnostic tools	Ethical implications of germline editing for cancer prevention and treatment	[271]
Developing new approaches to improve specificity of CRISPR-Cas9 gene editing	Exploring the use of CRISPR gene regulation to modulate gene expression	Testing the efficacy of CRISPR-based cancer therapy in combination with other standard therapies	Development of international regulatory frameworks for gene editing technology	Education and public outreach to promote understanding and acceptance of gene editing technology	[272]
Studying the impact of CRISPR gene editing on tumor microenvironment and the immune system	Developing CRISPR-based tools for non-invasive cancer detection and monitoring	Incorporating CRISPR gene editing into patient stratification and clinical trial design	Collaboration among researchers to advance the understanding of CRISPR mechanisms and their role in cancer biology	Addressing issues of genetic discrimination and privacy concerns related to gene editing technology	[18]
Designing CRISPR gene editing systems for the treatment of rare or difficult-to-treat cancers	Advancing gene editing technology to target complex genetic abnormalities in cancer cells	Establishing international databases to promote sharing of CRISPR-based therapy data and protocols	Development of guidelines and standards for quality control and product safety for CRISPR-based therapies	Ensuring equitable access to CRISPR-based cancer therapy in diverse patient populations	[270]
Developing gene editing strategies to overcome resistance to standard cancer therapies	Integration of CRISPR technology with nanotechnology for enhanced delivery and targeting of cancer cells	Establishing public–private partnerships to accelerate the development of CRISPR-based cancer therapies	Collaboration between clinicians and basic researchers to optimize the design and delivery of CRISPR-based cancer therapies	Evaluation of the long-term safety and efficacy of CRISPR-based cancer therapy	[273, 274]

### Development of new delivery methods

Researchers continue to explore and optimize various delivery systems, bringing us closer to realizing the full potential of CRISPR technology in oncology [275]. The main challenges in CRISPR-based cancer therapy using adeno-associated viruses (AAVs) as viral vectors include achieving specific and efficient delivery of CRISPR components to tumor cells. To address these challenges, researchers are exploring various modifications to the AAVs to enhance tumor-targeting capabilities, increase cellular uptake, and evade the body's immune response [276]. Additionally, developing strategies to limit non-targeted site effects and optimize the dose and administration route are essential. Liposomes offer a promising approach to deliver CRISPR components, as they can encapsulate the CRISPR machinery, protecting it from degradation and improving stability [277]. Furthermore, liposomes can be modified with targeting molecules to specifically bind to cancer cells, enhancing their specificity [278]. To enhance efficacy, researchers are optimizing liposome size, charge, and surface modifications to improve cellular uptake and endosomal escape, ensuring efficient release of CRISPR components within the tumor cells [279]. Safety is paramount in CRISPR-based therapies using viral vectors like AAVs. Researchers must ensure that the modified AAVs do not cause unintended immune responses or integrate into the host genome at undesirable locations [280]. The use of tissue-specific promoters and target-cell-specific enhancers can limit non-specific site effects [281]. Furthermore, rigorous preclinical studies and clinical trials are necessary to assess the safety and efficacy of AAV-based CRISPR therapies [277]. Nanoparticles made of polymers or inorganic materials offer alternative approaches to deliver CRISPR components. These nanoparticles can be designed with different physicochemical properties, which may influence cellular uptake and release kinetics [244]. While liposomes have advantages in encapsulation and modification, other nanoparticles may provide better stability or have unique capabilities for targeted delivery [282]. Cell-penetrating peptides and exosomes have the potential to improve CRISPR delivery [254]. Researchers can explore surface modifications of these delivery systems to increase their tumor-specific binding and uptake [279]. Additionally, optimizing cargo loading and release mechanisms could enhance the precise editing of target genes while minimizing unwanted effects [283].

### Combination therapy

**Fig. 10:**
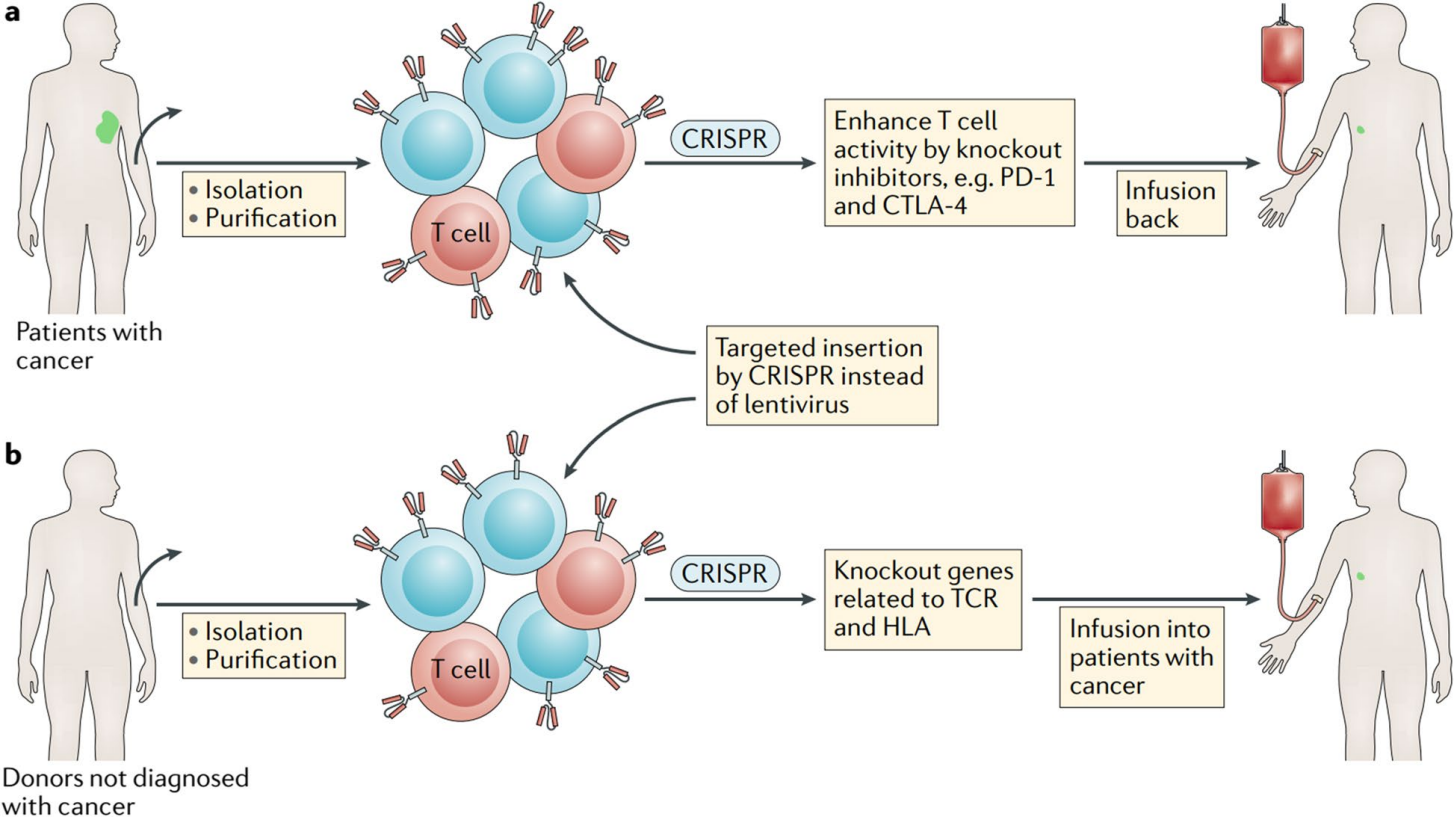
Illustrates the application of CRISPR in immuno-oncology. In scenario a, primary T cells extracted and purified from cancer patients can have a chimeric antigen receptor (CAR) inserted using CRISPR technology, instead of lentiviral-mediated transduction. CRISPR can also be employed to deactivate immune-checkpoint genes, such as PD-1 and CTLA-4, within these T cells. Alternatively, scenario b involves the isolation and purification of primary T cells from healthy donors not diagnosed with cancer. CRISPR systems are used to introduce a CAR into these cells, and they can also be utilized to inactivate the genes responsible for T cell receptor (TCR) and HLA components. This process generates 'universal' allogeneic CAR T cells, which can be infused into cancer patients. Reprinted from [15] with permission from Springer Nature

An encouraging approach involves merging CRISPR-based gene editing with other cancer treatments like immunotherapy or chemotherapy [284]. Preclinical investigations have demonstrated promising outcomes by combining CRISPR-based gene editing with immunotherapy or chemotherapy in cancer treatment. Figure 10 illustrates the application of CRISPR in immuno-oncology. For example, deactivating the PD-1 gene using CRISPR-Cas9 within cancer cells has led to a significant increase in the population of cancer-killing immune cells [285]. Additionally, the application of CRISPR-Cas9 alongside chemotherapy has shown potential in targeting chemotherapy-resistant cancer cells [285]. The combination of CRISPR-based gene editing with immunotherapy or chemotherapy offers several potential benefits. Firstly, it may lead to improved treatment efficacy as CRISPR can target specific genetic mutations associated with cancer, enhancing the precision of cancer treatment [286]. Secondly, combining CRISPR with immunotherapy can boost the body's immune response against cancer cells, potentially increasing the chances of tumor regression [287]. Thirdly, using CRISPR alongside chemotherapy can overcome drug resistance, improving the effectiveness of chemotherapy in combating cancer cells [288]. Currently, CRISPR-based combination therapies are primarily in preclinical stages, and their effectiveness in humans remains to be fully demonstrated [284]. In the future, advancements in delivery mechanisms and precision gene editing techniques may address some of these limitations, making CRISPR-based combination therapies a more viable option for cancer treatment [286]. Rigorous clinical trials are essential to validate the safety and efficacy of these approaches before they can be implemented in standard cancer care [287].

### Targeting multiple genes

Targeting multiple genes using CRISPR-based gene editing shows immense promise in complex cancer treatments, offering potential benefits in tumor regression and combating drug resistance [289]. However, it also raises safety concerns and faces challenges in clinical translation. Research efforts should continue to optimize and refine this technology for the potential benefit of cancer patients worldwide [290]. CRISPR-based gene editing has shown promising capabilities in targeting multiple genes simultaneously for cancer treatment [289]. Researchers have demonstrated its effectiveness in preclinical trials for lung cancer, where it targeted multiple commonly mutated genes using CRISPR-Cas9, resulting in tumor regression. This suggests that CRISPR technology has the potential to address complex genetic mutations that contribute to cancer development [290]. CRISPR-Cas9 has been successfully employed to combat drug resistance in cancer cells by simultaneously targeting multiple genes. By editing the genes responsible for drug resistance, researchers have enhanced cancer cells' sensitivity to chemotherapy, providing a potential solution to drug-resistant cancers [291]. While CRISPR-based gene editing shows promise, there are some safety concerns to consider. non-selective site effects, where CRISPR-Cas9 inadvertently edits unintended genes, could result in unforeseen consequences [292]. Extensive research and stringent safety measures are necessary to minimize such risks and ensure the safe application of CRISPR technology in cancer treatments [293]. The CRISPR-based gene editing's effectiveness in targeting multiple genes may vary between different types of cancer. Each cancer type is characterized by unique genetic mutations, necessitating tailored approaches [294].

### Personalized medicine

CRISPR-based gene editing presents a promising avenue for personalized cancer therapies [295]. However, thorough evaluation of its technical, ethical, and accessibility aspects is crucial to harness its potential safely and effectively for the benefit of patients [295]. CRISPR-based gene editing is a revolutionary tool that allows scientists to precisely modify specific genes in an organism's DNA [296]. CRISPR-Cas9, the most well-known system, uses a guide RNA to target a specific gene, and the Cas9 enzyme acts as molecular scissors to cut the DNA at that location. This break can then be repaired, leading to either gene knockout or precise gene editing [297]. In personalized cancer therapies, CRISPR-Cas9 can be utilized to target and correct genetic mutations responsible for cancer development. By identifying the specific genetic mutations causing cancer, scientists can design a customized approach to correct or disable these mutations, effectively halting cancer growth [298]. While CRISPR-based gene editing holds immense promise for personalized medicine, several challenges and risks must be carefully evaluated [295]. Off-target effects are a major concern, where CRISPR may unintentionally edit other parts of the genome, potentially leading to new health issues or promoting cancer development [299]. Ensuring the specificity and accuracy of CRISPR targeting is a critical aspect of its safe application [300]. Additionally, the delivery method of CRISPR components into the body needs to be optimized to ensure efficient targeting of cancer cells without causing unnecessary damage to healthy tissues [301]. Ethical considerations, such as germline editing, should also be thoroughly debated and regulated to avoid unintended consequences [297]. As of the current date, several CRISPR-based personalized cancer therapies are in various stages of preclinical and clinical trials [298]. While the successful preclinical studies in retinoblastoma and leukemia are promising, it's essential to understand that the transition from preclinical to clinical settings can present new challenges [299]. Rigorous clinical trials are necessary to assess the safety and efficacy of these therapies in human patients [295]. To make CRISPR-based personalized cancer therapies widely accessible, several factors need consideration [301]. First, research and development efforts should focus on optimizing the efficiency, accuracy, and safety of the CRISPR system [300]. Streamlining the manufacturing and delivery processes of CRISPR components could also reduce costs and increase accessibility [298]. Additionally, collaborations between academia, industry, and regulatory authorities can facilitate the translation of research findings into approved therapies [295]. To ensure equitable access, policymakers and healthcare providers need to work together to develop strategies for integrating personalized medicine, including CRISPR-based therapies, into existing healthcare systems [300].

### Synthetic lethality

CRISPR-based synthetic lethality holds promise as an innovative cancer treatment strategy, offering a more targeted and potentially effective approach to combatting cancer [302]. However, further research and clinical trials are necessary to fully evaluate its safety and efficacy before it can be widely implemented in cancer treatments [303]. Synthetic lethality refers to a phenomenon where the simultaneous disruption of two or more specific genes leads to the death of targeted cancer cells, while sparing normal cells [304]. In the context of CRISPR-based gene editing, this approach involves using CRISPR-Cas9 to simultaneously target two genes that are frequently mutated in cancer, exploiting the cancer's genetic vulnerabilities [305]. Synthetic lethality-based cancer treatments have the advantage of selectively targeting cancer cells with specific gene mutations, reducing the risk of harming healthy cells. This approach can potentially lead to more effective and precise therapies with fewer side effects than conventional treatments like chemotherapy and radiation [304]. Researchers have targeted gene combinations such as BRCA1 and BRCA2, frequently found mutated in breast and ovarian cancer, and PARP1 and BRCA1, commonly mutated in breast cancer [303]. By disrupting these gene pairs simultaneously, they trigger synthetic lethality in cancer cells [302]. Experimental studies have demonstrated the potential of CRISPR-based synthetic lethality as a cancer treatment strategy [304]. By targeting specific gene combinations in cancer cells, researchers have observed significant reductions in tumor growth and cell viability in preclinical models, indicating its potential as a promising therapeutic approach [303]. One major challenge is the delivery of CRISPR components to the tumor site efficiently [302]. Ensuring precise targeting and non-selective site effects are also important concerns. Additionally, identifying suitable gene combinations for specific cancer types and ensuring safety during clinical translation are vital considerations [303].

### CAR-T cell therapy

**Fig. 11:**
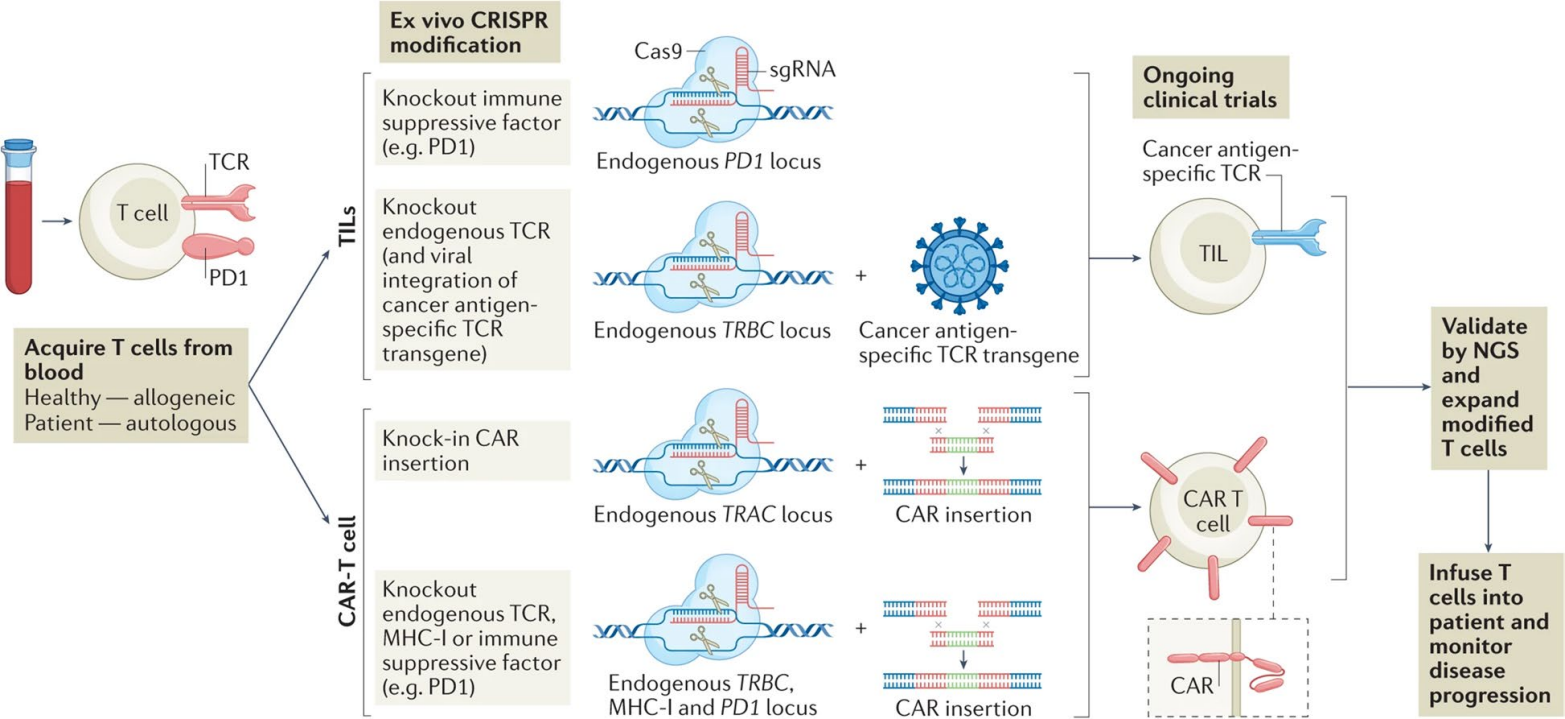
The ex vivo CRISPR manipulation of human T cells for adoptive T cell therapy. Ongoing clinical trials are currently dedicated to assessing the safety and effectiveness of CRISPR-engineered T cells through ex vivo modification and subsequent transfer. The goal is to enhance the anti-cancer response of T cells taken from healthy donors or patients. These trials investigate the potential of both allogeneic (from different donors) and autologous (from the patient themselves) T cells in various approaches, including tumor-infiltrating lymphocytes (TILs) and chimeric antigen receptor (CAR) T cells. One of the methods involves using CRISPR-Cas9 to remove immunosuppressive factors, like the programmed cell death protein 1 (PD1) ligand, from human primary T cells. This approach is being tested for adoptive T cell therapy involving both TILs and CAR T cells. The delivery of CRISPR-Cas9 ribonucleoproteins (RNPs) allows precise editing of immunosuppressive factors such as PD1 by guiding Cas9 to specific locations. Researchers are also exploring the deletion of the endogenous T cell receptor (TCR) using CRISPR-Cas9 to prevent TCR priming or immune rejection in the case of allogeneic T cells. Another avenue being explored is the replacement of the endogenous TCR with a cancer antigen-specific TCR, either through a TCR transgene or a CAR element. This has been shown to enhance the killing of cancer cells by T cells. In clinical trials, CRISPR-Cas9 homology-directed repair (HDR)-mediated knock-in to the T cell receptor α-chain constant (TRAC) locus is used to deliver CAR elements, and its efficacy is being tested. Additionally, CRISPR is used to delete the endogenous T cell receptor-β constant (TRBC) locus and endogenous major histocompatibility complex class I (MHC-I) to prevent immune rejection after transplant, and to remove immunosuppressive factors, all aimed at improving T cell activity in CAR T cells. Next-generation sequencing (NGS) is employed to confirm the engineered T cells, ensuring accurate on-target editing with minimal off-target effects. The expanded and validated T cells are then transplanted into the cancer patient, and disease progression is closely monitored to assess the safety and efficacy of the engineered T cells. Reprinted from [11] with permission from Springer Nature

CAR-T cell therapy involves modifying a patient's T cells using genetic engineering techniques to express chimeric antigen receptors (CARs) on their surface. These CARs enable T cells to recognize and bind to specific proteins, or antigens, present on cancer cells, leading to their destruction [306]. CRISPR-based gene editing offers the possibility to precisely modify T cells, enhancing their targeting capabilities [307]. By using CRISPR-Cas9, specific genes can be altered or inserted into T cells, enabling them to recognize and attack a particular protein expressed on various cancer cell types. This approach increases the efficiency and effectiveness of CAR-T cell therapy [308]. CAR-T cell therapy faces challenges such as cytokine release syndrome (CRS) and neurotoxicity, which are immune-mediated side effects resulting from the activation of T cells [309]. Managing these adverse events is crucial for the safe and successful implementation of CAR-T cell therapy [310]. Additionally, manufacturing CAR-T cells on a large scale and at a reasonable cost remains a challenge [307]. CAR-T cell therapy has shown remarkable success in certain types of blood cancers, such as acute lymphoblastic leukemia and non-Hodgkin lymphoma [310]. Clinical trials have reported high response rates and even durable remissions in some patients [308]. However, its effectiveness in solid tumors is still a significant area of research and development [307]. Long-term side effects of CAR-T cell therapy are not yet fully understood, as the therapy is relatively new [311]. However, some potential concerns include the persistence of CAR-T cells in the body, potential non-selective site effects of genetic modifications, and the impact on normal immune function [312]. To address manufacturing challenges, efforts are underway to optimize and streamline the production process, including automation and reducing the time and cost involved [308]. Furthermore, establishing specialized centers equipped with expertise and infrastructure can help address logistical challenges associated with CAR-T cell therapy [307]. Current limitations include the high cost of treatment, limited accessibility due to specialized requirements, and the need for personalized manufacturing for each patient [310]. Additionally, the effectiveness of CAR-T cell therapy can be influenced by factors such as antigen escape, tumor heterogeneity, and the immunosuppressive tumor microenvironment [313]. Figure 11 illustrates the ex vivo CRISPR manipulation of human T cells for adoptive T cell therapy.

### Combination of CRISPR-based gene editing with stem cell therapy

The combination of CRISPR-based gene editing with stem cell therapy has shown promising results in treating genetic diseases, such as sickle cell anemia [314]. By using CRISPR-Cas9 to correct the specific genetic mutation responsible for the disease in hematopoietic stem cells, researchers have been able to produce corrected blood cells [315]. The transplantation of these corrected stem cells into the patient's body has demonstrated potential in restoring healthy blood cells, alleviating the symptoms of sickle cell anemia [316]. The use of CRISPR-Cas9 in stem cell therapy raises concerns about non-selective site effects, where unintended genetic modifications may occur [314]. Ensuring the accuracy of CRISPR-Cas9 editing is crucial to prevent potential adverse consequences [316]. Researchers need to thoroughly evaluate and validate the specificity of the gene-editing process before proceeding with transplantation [317]. Additionally, long-term studies are necessary to monitor the stability of corrected stem cells and any potential unintended effects on the patient's health [316]. The scalability of this approach depends on several factors, including the ease of gene editing, the availability of patient-specific stem cells, and the ability to produce sufficient quantities of corrected cells for transplantation [314]. Advances in CRISPR technology and stem cell research are continuously improving scalability [318]. However, challenges such as efficient delivery of CRISPR components into stem cells and the cost of personalized treatments may limit its widespread implementation [314]. The use of CRISPR-based gene editing in stem cell therapy raises ethical considerations. Concerns include the potential for unintended genetic changes that could affect future generations if germ cells are edited [315]. Researchers must adhere to strict ethical guidelines and regulations to ensure that gene editing is conducted responsibly, with full transparency and informed consent from patients participating in clinical trials [317]. The combination of CRISPR-based gene editing with stem cell therapy holds promise for treating a wide range of genetic diseases beyond sickle cell anemia [319]. Disorders caused by single-gene mutations, such as cystic fibrosis and certain types of muscular dystrophy, could be potential targets for this approach [317]. However, each disease presents unique challenges and requires careful evaluation to determine its suitability for CRISPR-based gene editing and stem cell therapy [315].

### Combination of CRISPR-based gene editing with epigenetic therapy

The combination of CRISPR-based gene editing with epigenetic therapy allows for a more targeted and precise treatment approach [320]. While CRISPR can directly modify specific DNA sequences, epigenetic therapy can alter gene expression patterns without changing the underlying DNA sequence. By using both techniques in tandem, researchers can enhance the therapeutic effects, as CRISPR provides accurate gene targeting, and epigenetic therapy ensures sustained and controlled gene activity modifications [321]. One significant challenge is ensuring the safe and efficient delivery of CRISPR components and epigenetic drugs to target cells. Scientists must develop reliable delivery systems that can effectively penetrate the cells without causing non-selective site effects [322]. Additionally, maintaining long-term regulation of gene activity via epigenetic therapy might be challenging due to cellular processes that could revert these changes over time. Researchers need to develop strategies to maintain stable and heritable epigenetic modifications [323]. Genetic heterogeneity, where different cells within a tumor or disease exhibit distinct genetic mutations, poses a challenge for targeted therapies [256]. The combination of CRISPR and epigenetic therapy allows researchers to target specific mutations while bypassing others [324]. CRISPR can be programmed to recognize and edit particular mutations, while epigenetic therapy can suppress the activity of specific mutated genes, leading to a more comprehensive and effective treatment [325]. The combination of these powerful technologies raises ethical questions about potential non-selective site effects, unintended consequences, and germline editing [325]. Researchers and policymakers must ensure strict adherence to safety protocols and responsible use to prevent unintended genetic alterations [326]. Additionally, equitable access to such therapies and potential disparities in healthcare must be addressed to avoid exacerbating social inequalities [327]. As with any emerging technology, there are limitations to consider. The delivery of CRISPR components and epigenetic drugs to specific tissues or organs can be challenging [328]. Ongoing research focuses on refining delivery methods and increasing targeting efficiency [325]. Moreover, understanding the long-term consequences of epigenetic modifications and potential off-target effects remains a priority for further investigation to ensure the safety and efficacy of this combination therapy [327].

### Identification of new drug targets

CRISPR-based gene editing presents a valuable approach for discovering novel drug targets [329–331]. By deliberately deleting or modifying genes within cancer cells, scientists can observe which genes are vital for the growth and survival of these cells [332]. Various genes have been targeted using CRISPR-Cas9 in cancer cells to identify potential drug targets. Examples include oncogenes such as MYC, KRAS, and EGFR, as well as tumor suppressor genes like TP53 and PTEN [333]. The effects of gene deletion or modification in cancer cells using CRISPR-Cas9 are typically assessed by monitoring the cells' ability to grow and survive [48]. Researchers may compare the growth rates of cells with specific genes deleted or modified to those of unaltered control cells [333]. Additionally, cell viability assays and molecular analyses can provide insights into the impact of gene alterations on cellular functions and signaling pathways [329]. The importance of a gene for cancer cell growth and survival is typically determined by evaluating the impact of its deletion or modification on cell viability and proliferation [330, 333–336]. If the loss or alteration of a gene significantly impairs the cells' ability to grow and survive, it suggests that the gene plays a vital role in supporting cancer cell functions [332]. The identification of crucial genes using CRISPR-Cas9 provides valuable insights into the vulnerabilities and dependencies of cancer cells [48, 333, 335]. Genes found to be essential for cancer cell growth and survival can be further investigated as potential drug targets [333]. Targeting these genes with drugs may disrupt critical cellular processes, leading to the selective killing or suppression of cancer cells while minimizing harm to normal cells [329, 331, 337, 338]. Despite its potential, CRISPR-based gene editing for drug target discovery faces several challenges [333]. Off-target effects, incomplete gene knockout, and functional redundancy within cellular pathways can complicate data interpretation [333]. Additionally, the translation of CRISPR-based findings into effective drug targets requires further validation through preclinical and clinical studies [48]. Ensuring the specificity, efficacy, and safety of drugs targeting newly identified genes is crucial for successful clinical implementation [331, 333, 339].

## Data Availability

Not applicable.
