# Correction: Comprehensive review of CRISPR‑based gene editing: mechanisms, challenges, and applications in cancer therapy

**DOI:** 10.1186/s12943-024-01961-9

**Published:** 2024-02-27

**Authors:** Mohammad Chehelgerdi, Matin Chehelgerdi, Milad Khorramian‑Ghahfarokhi, Marjan Shafieizadeh, Esmaeil Mahmoudi, Fatemeh Eskandari, Mohsen Rashidi, Asghar Arshi, Abbas Mokhtari‑Farsani

**Affiliations:** 1 Novin Genome (NG) Lab, Research and Development Center for Biotechnology, Shahrekord, Iran; 2 Young Researchers and Elite Club, Shahrekord Branch, Islamic Azad University, Shahrekord, Iran; 3 Division of Biotechnology, Department of Pathobiology, School of Veterinary Medicine, Shiraz University, Shiraz, Iran; 4 Department of Chemistry, Shahid Bahonar University of Kerman, Kerman, Iran; 5 Faculty of Molecular and Cellular Biology ‑Genetics, Islamic Azad University of Falavarjan, Isfahan, Iran; 6 Department Pharmacology, Faculty of Medicine, Mazandaran University of Medical Sciences, Sari, Iran; 7 The Health of Plant and Livestock Products Research Center, Mazandaran University of Medical Sciences, Sari, Iran; 8 Young Researchers and Elite Club, Najafabad Branch, Islamic Azad University, Najafabad, Iran; 9 Department of Biology, Nourdanesh Institute of Higher Education, Meymeh, Isfahan, Iran

**Correction: Mol Cancer 23, 9 (2024)**

**https://doi.org/10.1186/s12943-023-01925-5**

Following publication of the original article [1], it has come to the author's attention that this article cites their work in an incorrect fashion and at least the related part of the paper raises some concern about the integrity of the reported information.

In Table 3 on clinical trials of CRISPR-based therapy of the manuscript, the authors cite our study (Ref 202 in the article, https://doi.org/10.1016/j.omtm.2022.03.018) and claim they demonstrated HPRT1-KO in cancer cells as a treatment strategy. Unfortunately, this statement does not reflect our study. They demonstrated for the first time that non-viral knock-in of CD19-specific CAR into primary human T cells is possible and effective with non-viral dsDNA templates (e.g. related to Fig. 11 of the Molecular Cancer article). This study was completely pre-clinical and had no relation to HPRT1.

After this perplexing finding, the corresponding author took some time to check other references of Table 3 and discovered that other references (such as References 201, 198, 197 that he checked) were also wrong. Glancing at other references in Table 3, it seems the authors cited predominantly review articles instead of original articles and at least some of the content was completely misaligned to the topic (e.g. Ref 198 cites a review on transplantation and GvhD; no relation to cancer therapy). Some of the related content is also non-sensical, suggesting either inexperience by the person preparing the table or potentially the use of a flawed AI tool. The correct Table 3 is given below.

**Table 3:** Clinical trials of CRISPR-based cancer therapy

Cancer Type	Treatment Approach	Patient Population	Description	Advantages	Disadvantages	Ref
Metastatic melanoma	TCR/CAR-T therapy targeting NY-ESO-1	Patients with NY-ESO-1 + tumors who failed prior therapy	T cells were edited to express NY-ESO-1 TCR/CAR, infused back into patients	Target specificity, long-term persistence	Possible off-target effects, limited efficacy in some patients	NCT04420539
Non-Hodgkin's Lymphoma	CD19-targeting CAR-T therapy	Patients with refractory/relapsed NHL	T cells were edited to express CD19 CAR, infused back into patients	High response rate, durable response in some patients	Cytokine release syndrome, neurotoxicity, potential for tumor antigen escape	NCT03939026
Renal Cell Carcinoma	Biological: CTX130	All patients with relapsed or refractory Renal Cell Carcinoma	CTX130 for Renal Cell Carcinoma; Phase 1, Open Label	Targets RCC specifically, innovative CAR T-cell therapy	Early phase, potential for adverse events	NCT04438083
Glioblastoma	EGFRvIII-targeting CAR-T therapy	Patients with recurrent GBM expressing EGFRvIII	T cells were edited to express EGFRvIII CAR, infused back into patients	Target specificity, potential for durable response	Heterogeneity of tumor antigen expression, potential for off-target effects	NCT02209376
Non-Small-Cell Lung Cancer	Targeted genome editing of MUC1 gene	Patients with MUC1 + esophageal squamous cell carcinoma	CRISPR/Cas9 was used to target the MUC1 gene in tumor cells, followed by infusion of edited T cells	Specific targeting of oncogenic driver mutation	Off-target effects, limited efficacy in some patients	[2]
Acute Myeloid Leukemia	FT538-targeting CAR-T therapy	Patients with relapsed/refractory AML	the use of FT538, a gene-edited CAR-T therapy targeting CD33 and CD19, in patients with relapsed/refractory AML and multiple myeloma	Target specificity, potential for durable response	Cytokine release syndrome, neurotoxicity	NCT04614636
Cervical cancer	Targeted genome editing of HPV16 E6 gene	Patients with HPV16 + recurrent or metastatic head and neck cancer	CRISPR/Cas9 was used to target the HPV16 E6 gene in tumor cells, followed by infusion of edited T cells	Specific targeting of oncogenic driver mutation	Off-target effects, limited efficacy in some patients	[3]
Lung Cancer	Targeted genome editing of KRAS gene	Patients with advanced KRAS-mutant lung cancer	CRISPR/Cas9 was used to target the KRAS gene in tumor cells, followed by infusion of edited T cells	Specific targeting of oncogenic driver mutation	Off-target effects, limited efficacy in some patients	[4]
Acute Lymphoblastic Leukemia	CD19-targeting CAR-T therapy	Pediatric patients with relapsed/refractory ALL	T cells were edited to express CD19 CAR, infused back into patients	High response rate, durable response in some patients	Cytokine release syndrome, neurotoxicity	[5]
Solid Tumors	Targeted genome editing of CCR4 gene	Patients with advanced solid tumors	CRISPR/Cas9 was used to target the CCR4 gene in tumor cells, followed by infusion of edited T cells	Specific targeting of oncogenic driver mutation	Off-target effects, limited efficacy in some patients	[6]
Cholangiocarcinoma	Targeted genome editing of IDH1 gene	Patients with advanced IDH1-mutant cholangiocarcinoma	CRISPR/Cas9 was used to target the IDH1 gene in tumor cells, followed by infusion of edited T cells	Specific targeting of oncogenic driver mutation	Off-target effects, limited efficacy in some patients	[7]
Solid Tumors	Targeted genome editing of TP53 gene	Patients with advanced solid tumors	CRISPR/Cas9 was used to target the TP53 gene in tumor cells, followed by infusion of edited T cells	Specific targeting of oncogenic driver mutation	Off-target effects, limited efficacy in some patients	[8]
Myeloma	Integrated PD1-BCMA-CART	Patients with relapsed/refractory myeloma	Non-viral site-directed integrated PD1-BCMA-CART in adult patients	High response rate, durable response in some patients	Cytokine release syndrome, neurotoxicity	NCT05308875
Solid Tumors	Targeted genome editing of PD-1 gene	Patients with advanced solid tumors	CRISPR/Cas9 was used to target the PD-1 gene in tumor cells, followed by infusion of edited T cells	Enhances anti-tumor immunity by disrupting immune checkpoint pathway	Off-target effects, limited efficacy in some patients	[9]
Metastatic Breast Cancer	Targeted genome editing of MUC1 gene	Patients with advanced MUC1 + solid tumors	CRISPR/Cas9 was used to target the MUC1 gene in tumor cells, followed by infusion of edited T cells	Specific targeting of oncogenic driver mutation	Off-target effects, limited efficacy in some patients	[10]
Multiple Solid Tumors	Targeted genome editing of EGFR gene	Patients with advanced EGFR-mutant solid tumors	CRISPR/Cas9 was used to target the EGFR gene in tumor cells, followed by infusion of edited T cells	Specific targeting of oncogenic driver mutation	Off-target effects, limited efficacy in some patients	NCT05201910
Multiple Solid Tumors	CRISPR/Cas9-mediated knockout of TGF-β receptor II	Patients with advanced TGF-β-overexpressing solid tumors	CRISPR/Cas9 was used to knock out the TGF-β receptor II gene in tumor cells, followed by infusion of edited T cells	Specific targeting of oncogenic driver mutation	Off-target effects, limited efficacy in some patients	[11]
Solid Tumors	CRISPR/Cas9-mediated knockout of AXL	Patients with advanced AXL-overexpressing solid tumors	CRISPR/Cas9 was used to knock out the AXL gene in tumor cells, followed by infusion of edited T cells	Specific targeting of oncogenic driver mutation	Off-target effects, limited efficacy in some patients	[12]
Lymphoma Cells	CRISPR/Cas9-mediated knockout of CD7	Patients with relapsed/refractory CD7 + leukemia/lymphoma	CRISPR/Cas9 was used to knock out the CD7 gene in T cells, followed by infusion of edited T cells	Specific targeting of B-cell antigen	Off-target effects, limited efficacy in some patients	NCT04005053
Hematologic Malignancy, Solid Malignancy	Non Interventional, Observational	All patients	Observational study to assess adverse events, serious adverse events, and events of special interest related to CRISPR CAR T cellular therapy. Also, evaluates overall survival and duration of remission/response	Broad patient inclusion, potential for long-term data collection	Observational, may not provide direct treatment benefits	NCT06208878
Multiple Myeloma, Melanoma, Synovial Sarcoma	Biological: NY-ESO-1 redirected autologous T cells, Drugs: Cyclophosphamide, Fludarabine	All patients	Evaluates safety, manufacturing feasibility, and clinical responses of NYCE T cells in multiple cancers	Targets NY-ESO-1, a known cancer-testis antigen, potential for multiple cancer types	Terminated study, may have limited data on long-term efficacy and safety	NCT03399448
Neurofibromatosis Type 1, Tumors of the CNS	Diagnostic Test: Collection of Stem Cells	All patients	Observational study to identify mutations in NF1 genes and measure neuronal characteristics of derived neurons. Uses CRISPR/CAS9 for genetic modifications	Provides insight into genetic mutations and potential therapeutic targets	Observational, not treatment-focused	NCT03332030
B-cell Lymphoma, Non-Hodgkin Lymphoma, B-cell Malignancy	Biological: CTX112	All patients	Phase 1/2 study evaluating safety, efficacy, and response rates of CTX112 in B-cell malignancies	Potential new treatment option	Limited to early-phase study, potential side effects	NCT05643742
Relapsed/Refractory Multiple Myeloma	Biological: CB-011	All patients	Phase 1 study on CRISPR-edited anti-BCMA CAR-T cells, focusing on dose-limiting toxicities and overall response rate	Innovative use of CRISPR technology	Early-phase, potential unknown risks	NCT05722418
Esophageal Cancer	Other: PD-1 Knockout T Cells	All patients	Interventional study assessing the efficacy and adverse events of PD-1 knockout T cells in treating esophageal cancer	Could offer a new therapeutic pathway	Safety and long-term efficacy concerns	NCT03081715
Solid Tumor, Adult, EGFR Overexpression	Biological: TGFβR-KO CAR-EGFR T Cells	All patients	Phase 1 study on the safety and response rates to TGFβR-KO CAR-EGFR T cell therapy in EGFR-positive tumors	Targets a specific cancer marker, potentially improving efficacy	Phase 1 study, limited safety and efficacy data	NCT04976218
Acute Myeloid Leukemia, in Relapse or Refractory	Drug: CB-012	All patients	Phase 1 study on CRISPR-edited anti-CLL-1 CAR-T cells, focusing on dose-limiting toxicities and overall response rate	Offers a targeted approach for a difficult-to-treat cancer	Early-phase study with inherent risks of novel therapy	NCT06128044
Advanced Stage EBV Associated Malignancies	Drugs: Fludarabine, Cyclophosphamide, Interleukin-2	All patients	Phase 1/2 study evaluating PD-1 knockout EBV-CTLs for safety and efficacy in EBV-associated malignancies	Innovative immunotherapy approach	Unknown status, potential risks and side effects	NCT03044743
T Cell Lymphoma	Biological: CTX130	All patients	Phase 1 study evaluating safety and efficacy of CTX130 in T or B cell malignancies, including dose escalation and cohort expansion	Potential new option for lymphoma treatment	Early-phase, possible adverse effects	NCT04502446
Lymphoma, Non-Hodgkin, Relapsed/Refractory B-Cell Non-Hodgkin Lymphoma	Genetic: CB-010, Drugs: Cyclophosphamide, Fludarabine	All patients	Phase 1 study on CRISPR-edited anti-CD19 CAR-T cells for B-cell non-Hodgkin lymphoma, assessing toxicities and tumor response	Utilizes CRISPR technology for targeted therapy	Phase 1, risks of new treatment modalities	NCT04637763
Solid Tumors	Biological: CTX131	All patients	Phase 1/2 study on CTX131 in relapsed or refractory solid tumors, assessing adverse events and objective response rate	Investigates new treatment for solid tumors	Limited to early-phase trials, potential side effects	NCT05795595
B Acute Lymphoblastic Leukemia (B-ALL)	Drug: PBLTT52CAR19	All patients with B-ALL seeking remission	TT52CAR19 Therapy for B-ALL; Phase 1, Open Label	Targets B-ALL specifically, aiming for remission	Early phase with limited patient data	NCT04557436
CD5 + Relapsed/Refractory Hematopoietic Malignancies, CLL, MCL, etc	Biological: CT125A cells; Drug: Cyclophosphamide, fludarabine	All patients with CD5 + hematopoietic malignancies seeking treatment for relapse/refractory conditions	Safety and Efficacy of CT125A Cells; Early Phase 1, Open Label	Addresses multiple CD5 + malignancies, comprehensive treatment approach	Early phase, potential for unknown AEs	NCT04767308
Multiple Myeloma	Biological: CTX120	All patients with relapsed or refractory Multiple Myeloma	CTX120 Study for Multiple Myeloma; Phase 1, Open Label	Potential for durable response in MM treatment	Risks associated with novel therapy, limited data on long-term outcomes	NCT04244656
Acute Lymphoblastic Leukemia, Chronic Lymphocytic Leukemia, Non-Hodgkin Lymphoma	Biological: PACE CART19	All patients with CD19 + leukemia and lymphoma seeking alternative treatments	PACE CART19 for CD19 + malignancies; Phase 1, Open Label	Innovative CRISPR-edited T-cell therapy, broad application	Withdrawn status limits data availability	NCT05037669
Metastatic Non-small Cell Lung Cancer	Drug: Cyclophosphamide; Other: PD-1 Knockout T Cells	All patients with metastatic NSCLC seeking new therapeutic options	PD-1 Knockout T Cells for NSCLC; Phase 1, Completed with results	Demonstrated safety and preliminary efficacy	Specific to PD-1 + , limited to metastatic stage	NCT02793856
Relapsed/Refractory T-cell Acute Lymphoid Leukaemia	Biological: Cryopreserved BE CAR7 T cells	All patients with T-cell ALL seeking remission ahead of allo-SCT	Base Edited CAR7 T Cells for T-cell Malignancies; Phase 1, Open Label	Novel base editing approach, focused on remission	Early phase, potential unknown risks	NCT05397184
High Grade Ovarian Serous Adenocarcinoma, Stage III	Other: Biospecimen Collection, Laboratory Biomarker Analysis, Device: Lavage	Female patients with advanced ovarian cancer for diagnostic purposes	Lavage for Ovarian Cancer Diagnosis; Not Applicable, Terminated with results	Non-invasive diagnostic approach, early detection	Terminated, may indicate limited feasibility or efficacy	NCT03606486
B Cell Leukemia, B Cell Lymphoma	Biological: Universal Dual Specificity CD19 and CD20 or CD22 CAR-T Cells	All patients with B cell leukemia or lymphoma seeking new treatments	Universal Dual Specificity CAR-T for Leukemia and Lymphoma; Phase 1/2, Open Label	Dual specificity may increase efficacy	Unknown status, risks associated with broad application	NCT03398967
B Cell Leukemia, B Cell Lymphoma	Biological: UCART019	All patients with CD19 + leukemia and lymphoma seeking alternative therapies	UCART019 for CD19 + malignancies; Phase 1/2, Open Label	Utilizes universal CAR T cells, broad potential application	Unknown status, early phase with inherent risks	NCT03166878
Acute Myeloid Leukemia (AML)	Biological: Donor-derived CD34 + HSC with CRISPR/Cas9-mediated CD33 deletion, Drug: Gemtuzumab Ozogamicin	All patients with Relapsed/Refractory AML	Interventional study aiming to test the engraftment of gene-edited CD34 + HSC and assess dose-limiting toxicity	Potentially improves therapeutic index of CD33-directed therapy	Risk of toxicities as per CTCAE v5.0	NCT05662904
Leukemia, Myeloid, Acute	Genetic: VOR33	All patients previously participating in a VOR33 study	Observational study to evaluate long-term safety and efficacy of VOR33	Offers insight into long-term effects and efficacy	Limited by observational design	NCT05309733
Acute Myeloid Leukemia	Genetic: NTLA-5001	All patients enrolled in the study	Interventional study evaluating NTLA-5001's safety and effectiveness	Potential novel treatment for AML	Terminated, possibly indicating issues	NCT05066165
Nasopharyngeal Carcinoma	Diagnostic Test: EBV antibodies test, EBV DNA test	Male patients for screening	Observational study assessing diagnostic tests' predictive values and sensitivity	Could improve early detection	Specific to male patients, limiting broader applicability	NCT05447169
Advanced Breast Cancer	Drug: AJMUC1- PD-1 gene knockout anti-MUC1 CAR-T cells	All patients with Advanced Breast Cancer	Interventional study on the safety and efficacy of CAR-T cell therapy	Innovative approach for advanced cases	Potential adverse events and toxicities	NCT05812326
Non-hodgkin Lymphoma, B Cell	Biological: Autologous CD19-STAR-T cell, Drugs: Fludarabine, Cyclophosphamide	All patients with r/r B-NHL	Interventional study assessing adverse events, DLTs, and MTD	Targets B-cell lymphoma specifically	Phase 1/2, indicating early research stage	NCT05631912
Hormone Refractory Prostate Cancer	Biological: PD-1 Knockout T Cells, Drug: Cyclophosphamide, IL-2	Male patients with castration-resistant cancer	Observational study on the safety and efficacy of PD-1 Knockout T Cells	Potential new therapy for resistant cases	Withdrawn, questioning viability	NCT02867345
Advanced Hepatocellular Carcinoma	Procedure: Transcatheter arterial chemoembolization, Biological: PD-1 knockout engineered T cells	All patients with Advanced Hepatocellular Carcinoma	Interventional study to assess safety and response rate	Combines TACE with immunotherapy	Early phase research	NCT04417764
Invasive Bladder Cancer Stage IV	Biological: PD-1 Knockout T Cells, Drug: Cyclophosphamide, IL-2	All patients with Stage IV Invasive Bladder Cancer	Interventional study on safety and response rate	Targets advanced bladder cancer specifically	Withdrawn, raising concerns about feasibility	NCT02863913
Non Hodgkin's Lymphoma	Biological: TRAC and Power3 Genes Knock-out Allogeneic CD19-targeting CAR-T cell, Drugs: Fludarabine, Cyclophosphamide	All patients with r/r B-NHL	Interventional study evaluating adverse events, RP2D, and ORR	Innovative CAR-T cell therapy approach	Still in early phase research	NCT06014073
Metastatic Renal Cell Carcinoma	Biological: PD-1 Knockout T Cells, Drug: Cyclophosphamide, IL-2	All patients with Metastatic Renal Cell Carcinoma	Interventional study on the safety and tolerability of PD-1 Knockout T cells	Offers a new treatment avenue for metastatic cases	Withdrawn, suggesting potential issues	NCT02867332
Human Papillomavirus-Related Malignant Neoplasm	Biological: TALEN, Biological: CRISPR/Cas9	Female patients	Evaluates the safety and efficacy in treating HPV-related cervical intraepithelial neoplasia with TALEN and CRISPR/Cas9. Measures include adverse events, HPV DNA titers, and cervical cytological changes	Targeted intervention for HPV, potential for HPV eradication	Limited to female patients, early phase study	NCT03057912
Solid Tumor, Adult	Biological: anti-mesothelin CAR-T cells	All patients	Study focuses on adverse events and clinical responses to anti-mesothelin CAR-T cell infusions in mesothelin positive solid tumors	Directly targets tumor-specific antigen, potential for specific efficacy	Early phase study, outcomes and long-term effects uncertain	NCT03545815
Gastrointestinal Cancers	Drug: Cyclophosphamide, Fludarabine, Biological: TIL	All patients	Evaluates the maximum tolerated dose, efficacy, and safety of autologous lymphocytes with CISH gene knockout in metastatic gastrointestinal cancers	Innovative genetic engineering approach, targets multiple cancer types	Non-randomized, potential adverse events related to treatment	NCT04426669
Solid Tumor, Adult	Biological: Mesothelin-directed CAR-T cells	All patients	Studies adverse events and clinical responses to mesothelin-directed CAR T cells infusion in multiple solid tumors	Targets specific tumor antigen, potential for broad application	Early phase study, specific adverse events associated with CAR-T therapy	NCT03747965
Neoplasms, Pancreatic	Drug: GSK3145095, Pembrolizumab	All patients	Assesses adverse events and efficacy of GSK3145095 alone and in combination with pembrolizumab in advanced solid tumors. Includes extensive safety profiling	Combination therapy potential, broad patient inclusion	Terminated study, results may be limited	NCT03681951
B-cell Malignancies	Biological: CTX110	All patients	Evaluates the incidence of adverse events, objective response rate, and duration of response with CTX110 in relapsed/refractory B-cell malignancies	Targeted therapy for B-cell malignancies, potential for durable responses	Phase 1/2 study, adverse events as a primary consideration	NCT04035434
Leukemia, Lymphoma	Genetic: XYF19 CAR-T cell, Drugs: Cyclophosphamide, Fludarabine	All patients	Assesses adverse events and maximum tolerated dose of XYF19 CAR-T cells in CD19 + leukemia or lymphoma	Specific targeting of CD19 + cells, innovative CAR-T approach	Limited to leukemia/lymphoma, potential severe CAR-T related adverse events	NCT04037566
Non-Small-Cell Lung Cancer	Drug: Fludarabine, Cyclophosphamide, Biological: CISH Inactivated TIL	All patients	Phase I/II study assessing safety, efficacy, ORR, and PFS of CISH inactivated TILs in NSCLC treatment	Innovative use of TILs, potential for significant efficacy in NSCLC	Phase 1/2, potential adverse events from treatment	NCT05566223

## References

[1] Chehelgerdi M, Chehelgerdi M, Khorramian-Ghahfarokhi M (2024). Comprehensive review of CRISPR-based gene editing: mechanisms, challenges, and applications in cancer therapy. Mol Cancer.

[2] Liu Q (2020). World-first phase I clinical trial for CRISPR-Cas9 PD-1-edited T-cells in advanced nonsmall cell lung cancer. Glob Med Genet.

[3] Zhen S, Li X. Oncogenic human papillomavirus: application of CRISPR/Cas9 therapeutic strategies for cervical cancer. Cell Physiol Biochem. 2018:2455–66. 10.1159/000486168 29268281

[4] Lu Y, Xue J, Deng T, Zhou X, Yu K, Deng L (2020). Safety and feasibility of CRISPR-edited T cells in patients with refractory non-small-cell lung cancer. Nat Med.

[5] Ottaviano G, Georgiadis C, Gkazi SA, Syed F, Zhan H, Etuk A, et al. Phase 1 clinical trial of CRISPR-engineered CAR19 universal T cells for treatment of children with refractory B cell leukemia. Sci Transl Med. 2022;14. 10.1126/scitranslmed.abq3010 36288281

[6] Khan A, Sarkar E. CRISPR/Cas9 encouraged CAR-T cell immunotherapy reporting efficient and safe clinical results towards cancer. Cancer Treat Res Commun. 2022. 10.1016/j.ctarc.2022.100641 36193597

[7] Crispo F, Pietrafesa M, Condelli V, Maddalena F, Bruno G, Piscazzi A, et al. IDH1 Targeting as a new potential option for intrahepatic cholangiocarcinoma treatment—current state and future perspectives. Molecules. 2020. 10.3390/molecules25163754 PMC7464324 32824685

[8] Mirgayazova R, Khadiullina R, Chasov V, Mingaleeva R, Miftakhova R, Rizvanov A, et al. Therapeutic editing of the TP53 gene: Is crispr/CAS9 an option? Genes (Basel). 2020:1–17. 10.3390/genes11060704 PMC7349023 32630614

[9] Chamberlain CA, Bennett EP, Kverneland AH, Svane IM, Donia M, Met Ö (2022). Highly efficient PD-1-targeted CRISPR-Cas9 for tumor-infiltrating lymphocyte-based adoptive T cell therapy. Mol Ther - Oncolytics.

[10] Bamdad CC, Yuan Y, Specht JM, Stewart AK, Smagghe BJ, Lin SC-M (2022). Phase I/II first-in-human CAR T–targeting MUC1 transmembrane cleavage product (MUC1*) in patients with metastatic breast cancer. J Clin Oncol..

[11] Alishah K, Birtel M, Masoumi E, Jafarzadeh L, Mirzaee HR, Hadjati J, et al. CRISPR/Cas9-mediated TGFβRII disruption enhances anti-tumor efficacy of human chimeric antigen receptor T cells in vitro. J Transl Med. 2021;19. 10.1186/s12967-021-03146-0 PMC8627098 34838059

[12] Hedrich V, Breitenecker K, Ortmayr G, Pupp F, Huber H, Chen D, et al. PRAME is a novel target of tumor-intrinsic Gas6/Axl activation and promotes cancer cell invasion in hepatocellular carcinoma. Cancers (Basel). 2023;15. 10.3390/cancers15092415 PMC10177160 37173882

